# Accounting for Sampling Error When Inferring Population Synchrony from Time-Series Data: A Bayesian State-Space Modelling Approach with Applications

**DOI:** 10.1371/journal.pone.0087084

**Published:** 2014-01-29

**Authors:** Hugues Santin-Janin, Bernard Hugueny, Philippe Aubry, David Fouchet, Olivier Gimenez, Dominique Pontier

**Affiliations:** 1 Office National de la Chasse et de la Faune Sauvage, Direction des Études et de la Recherche, Le Perray-en-Yvelines, France; 2 Université de Lyon, Lyon, Université Lyon 1, CNRS, UMR 5558, Laboratoire de Biométrie et Biologie Evolutive, Villeurbanne, France; 3 UMR Biology of Aquatic Organims and Ecosystems, MNHN-IRD-CNRS-UPMC, Muséum National d’Histoire Naturelle, Paris, France; 4 Centre d’Ecologie Fonctionnelle et Evolutive, CNRS, UMR 5175, Montpellier, France; H. Lee Moffitt Cancer Center & Research Institute, United States of America

## Abstract

**Background:**

Data collected to inform time variations in natural population size are tainted by sampling error. Ignoring sampling error in population dynamics models induces bias in parameter estimators, e.g., density-dependence. In particular, when sampling errors are independent among populations, the classical estimator of the synchrony strength (zero-lag correlation) is biased downward. However, this bias is rarely taken into account in synchrony studies although it may lead to overemphasizing the role of intrinsic factors (e.g., dispersal) with respect to extrinsic factors (the Moran effect) in generating population synchrony as well as to underestimating the extinction risk of a metapopulation.

**Methodology/Principal findings:**

The aim of this paper was first to illustrate the extent of the bias that can be encountered in empirical studies when sampling error is neglected. Second, we presented a space-state modelling approach that explicitly accounts for sampling error when quantifying population synchrony. Third, we exemplify our approach with datasets for which sampling variance (i) has been previously estimated, and (ii) has to be jointly estimated with population synchrony. Finally, we compared our results to those of a standard approach neglecting sampling variance. We showed that ignoring sampling variance can mask a synchrony pattern whatever its true value and that the common practice of averaging few replicates of population size estimates poorly performed at decreasing the bias of the classical estimator of the synchrony strength.

**Conclusion/Significance:**

The state-space model used in this study provides a flexible way of accurately quantifying the strength of synchrony patterns from most population size data encountered in field studies, including over-dispersed count data. We provided a user-friendly R-program and a tutorial example to encourage further studies aiming at quantifying the strength of population synchrony to account for uncertainty in population size estimates.

## Introduction

Observed in many taxa, spatial population synchrony is the tendency of spatially disjoint populations to exhibit correlated fluctuations [1]. Besides providing potential cues on the factors underlying population fluctuations [2]–[4] the spatial synchrony of local population dynamics can be a critical determinant of the stability and the persistence of metapopulations [5]. Since a complete census of a population is generally impossible, most abundance data used in empirical studies and *a fortiori* in synchrony studies arise from a sampling procedure that provides an estimate of the true population size tainted by sampling error. Exploring spatio-temporal patterns of variations in true population size from such data is challenging because ignoring sampling error may lead to biased estimation of some key population dynamic parameters such as the density-dependence strength [6], the temporal variability of population size [7] and all related parameters including population synchrony [8]. As a consequence, many studies have developed methods to account for sampling error when estimating density-dependence (e.g., [9]–[12]) and temporal variability in population size (e.g., [7], [13]). However, little attention has been given to the consequence of ignoring sampling variance when quantifying the strength of synchrony patterns (but see [8]).

The sampling strategy commonly used in synchrony studies consists in monitoring animal abundance/density on the same sites (i.e., areas defined geographically) at fixed time intervals, typically every year. Within each year, the mean population size, i.e., the mean number of individuals present at a given site, is estimated from the numbers of individuals counted (e.g., along transects) either simultaneously on several spatial sampling units, or repeatedly on the same spatial unit during a period sufficiently short to hold the population closed geographically and demographically. In ecological literature, the term *sampling error* is used in a broad sense to represent the difference between the true population size and its estimate, i.e., it mixes two types of errors [14], [15]: (i) the ‘pure’ sampling error arising because only parts of the study site are prospected and/or because the positions of animals change between sampling occasions [16], and (ii) the observation error arising because of imperfect detectability of individuals within the sampling units. The sampling error induces variability (called sampling variance) in the estimated population size that is not meaningful for population biologists. By contrast, the process variations, which are temporal variations in true population size, are of prime interest in synchrony studies. However, most of them ignore sampling error and thus quantify synchrony among temporal variations in population size estimates resulting from the combination of both process and sampling variations.

The approach widely used for quantifying the strength of the synchrony between two sampled populations [17] consists in computing a zero-lag correlation between the two time series of observed (log) population sizes (called the population correlation 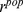). Statistical inference of population synchrony using this approach is difficult because of the serial correlation (i.e., temporal autocorrelation) often present in time series of population size [17]. To overcome this difficulty, an alternative approach based on population dynamics modelling is sometimes preferred [17]. It consists in (i) modelling local population using population dynamic models – where process and sampling variations are not separated (process-error-only models [10]) – to account for serial correlation through density-dependence and, (ii) computing zero-lag correlation between the residuals (called the process error correlation 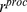). These two notions of spatial synchrony (population *vs.* process) are unified by the Moran theorem [18], which states that when two populations are subject to the same linear density-dependence and are not connected by dispersal, 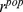
and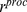 will provide identical results. In most cases the conditions underlying this equality are not fulfilled, but 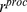 may nevertheless provides a useful information because, for non experimental populations, it is the best way for estimating the so-called Moran effect [3], i.e., the extrinsic, environmental component of synchrony. When dispersal is not explicitly accounted for in the model by deterministic coupling between populations, then 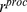
provides a rough estimate of environmental synchrony because it is generally less affected by dispersal than 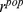 [19]. In sampled populations, 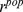
and 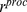
are underestimates of the theoretical values, 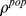 and 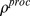, respectively. This is largely acknowledged [8], [20], [21] but has rarely been accounted for in previous works dealing with the synchrony of natural populations. Therefore, whether it is a source of concern remains to be assessed.

From a biological point of view, ignoring sampling variance not only leads to underestimation of population synchrony but can also lead to a misidentification of the underlying mechanisms. For instance, only climate can induce high synchrony between populations that are separated by large distances [22], but sampling error may lower the observed synchrony to a point where climate no more appears as the sole plausible mechanism. Another consequence of underestimating population synchrony is underestimation of the extinction risk of a metapopulation [5].

This inappropriate assessment of both statistical and biological consequences of ignoring sampling variance in synchrony studies may be explained by a lack of research exploring the size of the bias in synchrony estimation that can be encountered in sampled populations. The bias quantification would facilitate an understanding of the ways in which the sampling error influences an analysis that ignores it [23]. In this paper, we will use realistic values of sampling error, estimated from natural populations, to show how serious the problem could be. First, we use published estimates of sampling variance for bird species to show that the approach commonly used (i.e., using 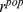) for quantifying population synchrony can lead to strong downward bias in synchrony estimations. Second, we focus on the quantification of population synchrony in a population modelling framework (i.e., using 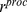). We present a state-space model for quantifying population synchrony that explicitly accounts for sampling variance and illustrate our approach using (i) a published dataset of fish (*Alestes baremoze*) abundance [24] for which sampling variance has been estimated in a prior study, and (ii) an original dataset of feral cat *(Felis silvestris catus*) abundance for which sampling variance has to be estimated. Then we compare our results to those obtained using a standard approach ignoring sampling variance. We deliberately used systems composed of few populations unlikely to be strongly connected by dispersal and simple linear models to account for their dynamics. Indeed, our aim is to focus on the consequences of sampling error on the estimation of population synchrony not to deal with all the factors known to be potentially relevant, such as non-linearity, dispersal, demographic stochasticity, heterogeneity among populations, biotic interactions, colour of the environmental noise and distance between sites.

## Materials and Methods

### 1. Inferring Population Synchrony

#### 1.1. Inferential framework, bias definition and notations

Suppose that we can observe without error the (biological) log population size *x_ij_* of some species at 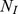 sites and 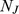 dates (typically years). Let 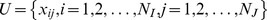 be the set of corresponding data (see File S1 for a list of the main mathematical notations used). 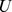 is a finite (statistical) population, and all statistics computed from *U* are fixed values called finite population parameters. This is the case of the correlation between two time series of log population sizes 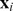 and 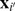 (two vectors of length 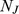), that we denoted 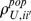, and of the correlation between the corresponding two time series of process errors 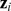 and 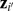, denoted 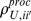 (see File S2). Statistical inference has no sense here unless we make reference to a stochastic process that may have generated the particular 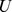 under consideration. Such a stochastic process is known as a superpopulation model, that is, a model able to generate an infinite set of possible populations 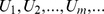 sharing some statistical features with 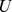 [25]. In population dynamic studies, the superpopulation model is usually called the “population process”. Under this framework, the values 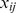 and 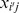 are viewed as realizations of random variables 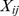 and 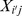, respectively. Thus, at the superpopulation level we consider two parameters: the correlation between 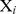 and 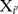, that we denoted 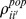 and the correlation between the corresponding process errors 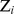 and 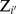 that we denoted 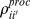
(see File S2).

At this stage, the statistical inference concerns the superpopulation parameters, estimated by their finite population counterpart, i.e., the inference concerns the parameter 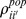
that is estimated by 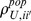. The source of stochasticity needed for the inference is the population process and is denoted in this paper using the subscript ‘p’ for the expectation, variance and covariance operators. In addition, the concept of (statistical) bias of an estimator may be denoted *p*-bias.

Now, we introduce another level of stochasticity by considering that the (biological) observed log population sizes are tainted by sampling errors. We do not know 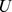 but only a set *s* of observed values 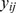 that differ from the true values 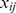
of 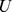 by some quantity that we called ‘sampling error’, generated by a sampling process. At the level of *s*, we can consider the correlation between two observed time series 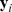 and 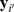 of length 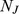, that we denoted 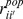, and the correlation between the corresponding two time series of residuals – obtained by fitting population dynamics models where process and sampling variations are not separated – denoted 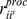.

As population and sampling processes are hierarchically related, two possible levels of inference can be considered. If the scope of the inference is 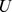, then the source of stochasticity comes from the sampling process and is denoted in this paper using the subscript ‘s’ for the expectation, variance and covariance operators. For instance, for descriptive purpose, we may want to estimate the finite population parameter 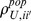 by using 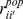. For scientific purpose, the relevant inference level is the superpopulation one (here the population process). With the two levels of statistical inference involved, 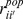 is an estimator of the finite population parameter 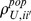 which is in turn an estimator of the superpopulation parameter 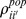. The possible bias of 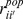 as an estimator of 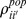 may be denoted as a *ps*-bias, which involves both *s*-expectation and *p*-expectation (see File S2 for details). In what follows we assessed the magnitude of the *ps*-bias of 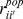 as an estimator of 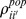 that can be encountered in natural populations.

#### 1.2. Bias of the usual synchrony estimator (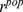) due to sampling error

The approach commonly used to quantify spatial synchrony between 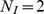 sampled populations consists in computing the zero-lag correlation:
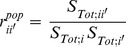
(1)where 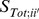
is the covariance measured between 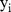 and 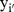; 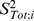
and 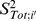
are the total temporal variances in observed log population size – the magnitude of the temporal variations in 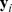 and 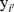 due to both population (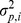) and sampling (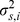) processes – for site *i* and *i’,* respectively.

In absence of sampling error, 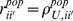 is a *p*-unbiased estimator of 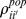 since 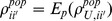. However, it can be shown that when sampling variance is constant (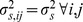) and sampling errors are additive and independent among populations, 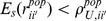 and therefore 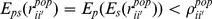, i.e., 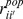
is a downward *ps*-biased estimator of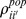. When an estimate 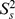 of the sampling variance 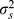 is available, the theoretical synchrony among populations can be estimated according to [24]:
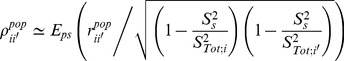
(2)


From eqn 2 it follows that the *ps*-bias of the correlation estimator 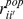 depends on the contribution of sampling variance to the total temporal variance in observed population size. Besides this statistical consideration, what is important from a methodological point of view is to assess the extent of the *ps*-bias that can be encountered in empirical studies when the sampling error is neglected. Based on both eqn 2 and published estimates of the ratio between sampling and total temporal variance, we illustrated the extent of the *ps*-bias that can be expected when quantifying synchrony among time series of observed population size. Link et al. [26] have estimated such a ratio for 98 bird species. We selected 9 species whose ratio spread over the range of the 98 ratio values. Based on these 9 ratio values, we used eqn 2 to compute the *ps*-bias that can be expected when quantifying synchrony among populations of these species using 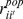, without accounting for sampling error.

#### 1.3. Quantifying population synchrony in the presence of sampling error

When (i) sampling variance is constant and has been estimated, and (ii) sampling errors are both additive and independent among populations, eqn 2 can be used to correct correlation estimates (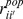) *a posteriori*. However, this case is unlikely to be frequently encountered in practice because sampling variance may depend on population size and hence is not constant through time, or the number of spatial or temporal sampling units used may have varied through time (e.g., from one year to the next) and, as a result, sampling variance too. To overcome these difficulties, we propose to quantify population synchrony in a more general framework of population dynamics modelling. The aim is to quantify density-dependence and synchrony between process errors (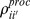) to in turn quantify the synchrony between populations (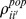). We consider the general case with 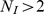, by introducing the average correlation among the process errors, which we denoted 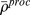, and the average correlation among populations, which we denoted 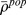 (see File S2 for details). State-space models provide a flexible way of accounting for sampling error when quantifying such population dynamic parameters [9]. In what follows, we present a Bayesian state-space model that allows estimating both density-dependence and synchrony among population processes – and in turn population synchrony – from time series of observed log population size. In a first stage we considered the case where sampling variance has been estimated in a prior study and, in a second stage, we considered the case where synchrony and sampling variance have to be jointly estimated.

When population size is estimated using, for example, distance sampling methods [27], we get an estimate (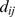) of the population density at site *i* and time *j* as well as a coefficient of variation estimate (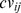) reflecting the uncertainty about 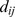. To account for this uncertainty when quantifying population synchrony between 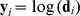 and 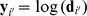, we propose to use a state-space framework where we can jointly define the sampling and the state processes. The state process models the underlying population dynamics that changes log population size over time and the sampling process links the unobservable true log population size to its estimate [9]. In our model, the estimates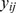 are viewed as normally distributed with mean 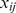
and variance 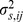. We considered the following system:
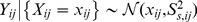
(3)

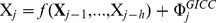
(4)where eqn 3 describes the sampling process with 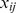 the unobservable true log population size at site *i* and time *j* to be estimated. In the example of distance sampling, the 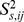-values are distance sampling estimates (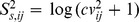) of the sampling variance (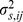) that play the role of assigned parameter values. Eqn 4 describes the state process, with 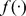 a function of past population states representing deterministic variations in local population dynamics. In its more general definition,
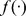
may accommodate for non-linear and/or heterogeneous population dynamics as well as for predation by nomadic predators or dispersal among populations. In eqn 4 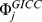
is a 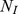 *-*element vector of stochastic terms (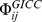) that describes how the process error variance (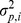) is partitioned into shared and unshared variations among sites (see Discussion in [28]) after accounting for deterministic variations in true population size:



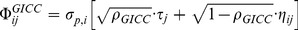
(5)In eqn 5, 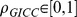 is the state-space model parameter – thereafter called Generalised Intra-Class Correlation – corresponding to the fraction of the residual process variance (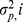) that is shared among sites, i.e., quantifying the average synchrony (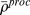) among residual process variations (see File S3); the random terms 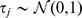 and 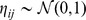 represent shared and unshared variations, respectively. We assume that the random variables 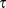 and 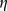 are mutually independent and that each is exchangeable between two times *j* and *j’*. The fitting method of the state-space model will determine the statistical properties of the different estimators such as 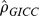. An alternative approach would be to use 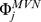 instead of 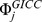, with 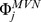 following a multivariate normal distribution of null mean vector and variance-covariance matrix 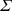 (see File S2). In this paper we used 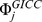 since it requires less parameters and enables the explicit modeling of the synchronous component of temporal variations in population sizes.

According to the superpopulation model (see File S2), when two populations are subject to the same linear density-dependence and are not connected by dispersal (i.e., when
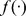
is simply a linear function of past population states), then 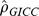 is also an estimator of the average population synchrony (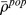). If 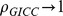, then the shared process pattern would account for a large fraction of the total temporal variance at each site; the time variations in abundance would then be synchronous among the populations. Conversely, if 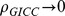, then the time variations in abundance are asynchronous among the populations. When 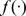 is non-linear, differs among populations, or includes dispersal or predation mechanisms, then 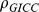 is no longer an estimator of 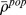. In this case, an estimate of the average synchrony among populations (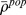) can be obtained through Monte Carlo simulations, or in some cases analytically, based on the model parameter estimates [29]. When the magnitude of temporal fluctuations are similar among sites – as such we can consider 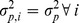 – an alternative parameterization of 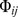
can be used: 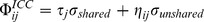, where 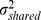 and 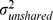 represent the magnitude of the shared and unshared fluctuations, respectively, in residual process variations among populations with 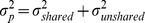. Like 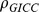 in eqn 5, the ratio 
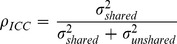
 (called Intra-Class Correlation) leads to an estimator 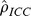 of the strength of the average synchrony among population processes 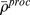
(see File S3). It follows that for populations that are not connected by dispersal and subject to the same linear density-dependence, 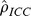
is also an estimator of the average synchrony among populations 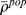. When the condition 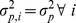 applies, 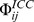 may be preferred to 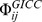 because of the reduced number of parameters involved. When 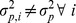 we recommend using 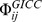 because 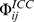
will lead to underestimate 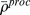. By definition both the intra-class correlation and the generalized intra-class correlation are devoted to the quantification of positive synchrony patterns which was the focus of most synchrony studies (for a review see [1]). However in some cases, for example in presence of apparent competition, a negative correlation between populations can be expected [30]. When residuals process variation are modeled using 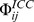 or 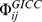, exploring such a pattern of negative correlation can still be revealed by quantifying the correlation matrix between the 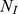 time series of estimates of residuals process variations 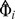.

The state-space modelling approach presented above can be extended to accommodate the case where sampling variance and population synchrony have to be jointly estimated. This typically applies when the collected data (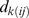) are densities of individuals present on 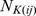 spatial or temporal sampling units (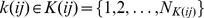) nested within site *i* at time *j*. Here we consider that the number of spatial or temporal sampling units may have varied through sites and/or time. Updating eqn 3, the sampling process becomes:
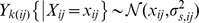
with 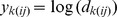 and where 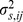 is here a parameter to be estimated.

Bayesian methods provide a flexible way of fitting both types of model. Generally, if the data are informative enough, the likelihood dominates the non-informative priors and the results are close to that of a frequentist inference [31]. In the following analysis, summaries of the posterior distribution of the parameters are obtained using the Markov Chain Monte Carlo (MCMC) algorithms implemented in the JAGS 3.3 software [32]. We used the R-packages dclone [33] and RJAGS [34] to call JAGS from the R 3.0.1 software [35].

### 2. Illustrative Examples

We aimed to illustrate how the state-space model presented in the previous section can be used to account for sampling variance when quantifying the strength of population synchrony from time series of observed population size. In addition, we performed a ‘what-if’ scenario analysis to exemplify the impact of neglecting sampling variance when quantifying population synchrony, i.e., we compared the results obtained with our state-space model with those of a standard approach neglecting sampling error. We considered first the case where sampling variance has been estimated in a previous study; in a second stage, we considered the case where both sampling variance and spatial synchrony have to be estimated. In both examples, the populations are suspected to be synchronised by a Moran effect.

#### 2.1. Estimation of spatial synchrony using an independent estimate of sampling variance

In this example, we used the abundance data analysed by Tedesco et al. [24] to explore intra- and inter-specific synchrony patterns in population dynamics of four West African fishes caused by a Moran effect. The only topic we considered here is the way the sampling error is accounted for, i.e., we were not interested in improving other aspects of the analysis performed by Tedesco et al. [24]. We used 24-year time series (1974–1997) of abundance estimates of *Alestes baremoze* collected at three sites (inter-site distances: 176–367 km) in two different catchment basins in Côte d’Ivoire (West Africa). On a given site *i* within a given year *j*, several gill-net fishing trials were conducted at a few month intervals. Note that the numbers 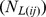 and the dates of the experimental fishing (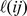) changed according to site and year. As there is only one fish abundance estimate per experimental fishing (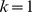), in what follows we omitted the index 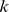 that denoted the replicates. The data are expressed as catch per unit effort (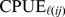), which is the number of fish caught in 100 m^2^ of net per night at a given site *i* during a given year *j* and a given experimental fishing 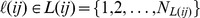. Since the sampling variance cannot be estimated with the data available from Côte d’Ivoire samples, it was estimated from data of the same species from similar river systems (Mali-Guinea) and sampled with the same technique. Assuming that it is constant on the log scale, Tedesco et al. [24] reported the following estimate 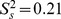 of sampling variance (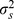) for one experimental fishing 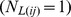.

**Figure 1:**
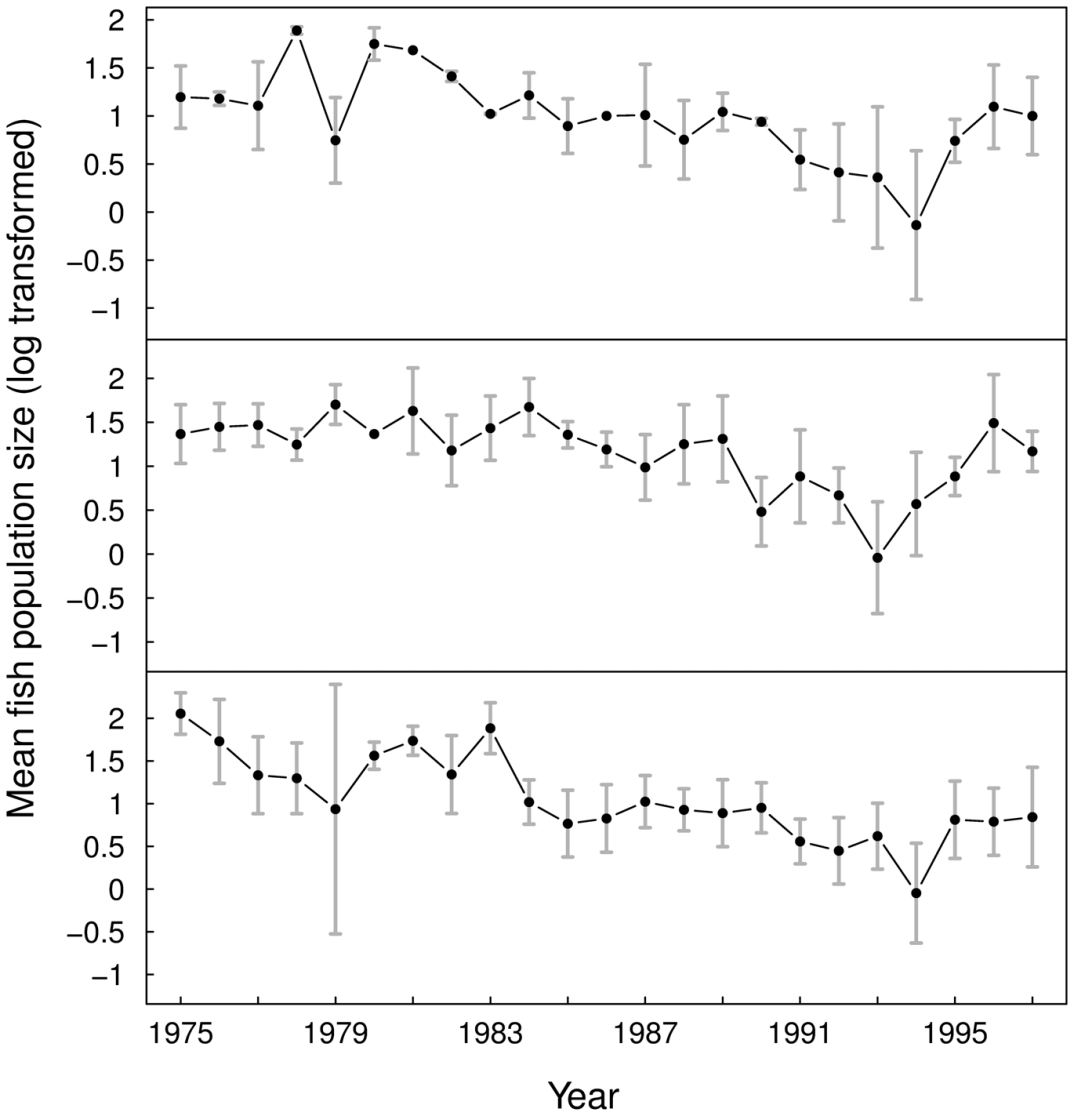
Averaged time variations in observed fish population size at the three study sites. Points represent the mean of log-transformed fish population size estimates. The grey bars represent the respective standard deviation of the observed means on the logarithmic scale.

Since we are interested in quantifying synchrony among inter-annual variations in mean population size, the difference in the average abundance of fish between the experimental fishing at each time *j* at site *i* was considered to be part of the sampling process:
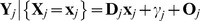



with 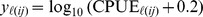 where 0.2 is the minimal non-zero value found in the series [following 24]; 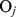 is a 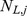-element vector (
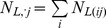
) of random variables 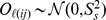 where 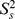 plays the role of assigned parameter value; 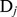 is an 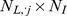 matrix of 0 s and 1 s that translates the 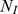 true log population size at time *j* into 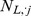 true log population size at time *j*; and 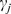
is a 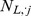-element random vector with 
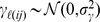
 that accounts for the difference in the average abundance of fish between the experimental fishing within each year *j* at site *i*. Following Tedesco et al. [24], we used a stochastic Gompertz model to describe the deterministic inter-annual dynamics of the fish populations. Since the magnitudes of the temporal variations of fish abundance are similar among the study sites (see Fig. 1), we considered the following state process to quantify the strength of synchrony among fish populations:
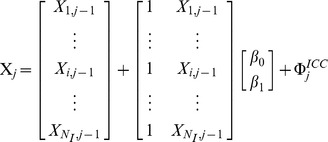
where 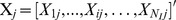 is the true mean (log) number of catch per unit effort at site *i* in year *j*; 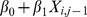 is the first order Gompertz model where 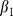 is a coefficient of density-dependence and 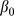 is an intercept. Here 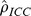 is an estimator of the strength of the average synchrony (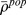) among fish populations (see Materiel and Methods section 1.3). Both the fish dataset and the R-program used to fit the state-space model are available as supplementary file (File S4)

#### 2.2. Joint estimation of spatial synchrony and sampling variance

Here, we aimed at illustrating (i) the use of the Generalised Intra-Class Correlation instead of Intra-Class Correlation to estimate average population synchrony, and (ii) the joint estimation of spatial synchrony and sampling variance using count data. Thus, for the sake of simplicity, we considered the same density-dependence structure as in the previous example. We used an original dataset of feral cat abundance collected at four sites (Port-aux-Français, Port-Jeanne-d’Arc, Port-Couvreux, Ratmanoff; inter-site distances: 20–60 km) of the Grande Terre Island of the Kerguelen Archipelago (sub-Antarctic) from 1996 to 2007 [36], [37]. The sampling protocol used is based on the collection of replicated count data (see File S2) within field sessions of 7–10 days all year round. Based on the climate dynamics, we can distinguish two seasons per year: (1) ‘Summer’ from November to April and (2) ‘Winter’ from May to October. At each site, a permanent linear transect delimited by coloured posts was established. Typically, during a field session at a given site *i* and during a given time *j* (corresponding to a given season in a given year), the transect was covered several times by a single trained observer. Each time the transect was travelled, the total number of adult cats detected by the observer on either side of the transect was recorded. Each site was visited approximately every three months, but due to climatic and logistical constraints, the order and the frequency of the visits changed from one season to the next, i.e., there was a varying number of field sessions within each site-time and varying number of counts within each field session. Therefore, the data are expressed as the total number of adult cats (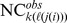) observed during a given count (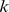) in a given field session (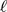), at a given time (*j*) at a given site (*i*). Note that in this case study *j* is nested in *i*, 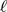 is nested in *j*, and *k* is nested in 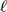. To avoid cumbersome notation we did not report the “nested” notation, i.e., we used 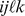 instead of 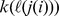. On average, the transects were covered 21 times per site-time (min = 2, max = 69) and overall, 1496 transect counts (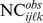) were performed during the study period.

It is largely acknowledged that exploring spatio-temporal pattern of variation in population size from such “raw” count data requires consideration for imperfect detection in order to prevent any confounding effect of detectability on abundance [38]. By contrast to strip-transect counts, which are complete census within the strip-area, it is likely that the observer only detect a fraction of the actual number of cats present in the strip. To account for imperfect detection in our state-space model we follow the approach of Kéry et al. [39] which consists in defining an observation process:
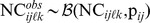
where 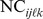
is the true number of cats and 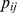 is the cat detection probability at site *i* at time *j*. We showed elsewhere that the relationship between the mean and the variance of the 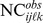 has a quadratic form [40] leading us to assume that the distribution of the number of cats available for detection within the field sessions can be approximated by a negative binomial distribution (e.g., [41] pp 185–220) with mean 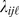 and variance 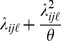, where 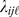 is the theoretical mean number of cats present during the 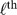 field session at time *j* at site *i*, and *θ* a is an unknown constant independent of *λ* that accounts for the over-dispersion of the data in comparison with the Poisson model. As for the fish dataset, the difference in the average abundance of cats between the counting sessions (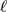) within each time *j* at site *i* was considered to be part of the sampling process:
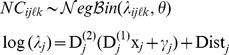
(6)with the total numbers of observations and field sessions at time j given by 
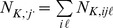
 and 
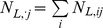
, respectively. In eqn 6, 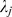 is a 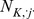-element vector of 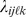; 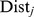 is a 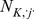-element vector containing the logarithmic distance covered by the observer during a given count (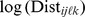) to account for the likely increase in the number of cats available for detection with the distance covered; 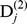 and 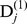 are two 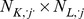 and 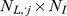 matrices of 0 s and 1 s that translate the 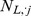 (log) mean population size at time j into 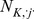 (log) mean population size at time j and the 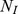 (log) mean population size at time j into 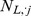 (log) mean population size at time j, respectively; and 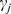 is a random vector with 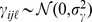 a random term that accounts for the difference in the (log) mean abundance of cats between the field sessions (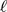) within each time j at site i.

**Figure 2:**
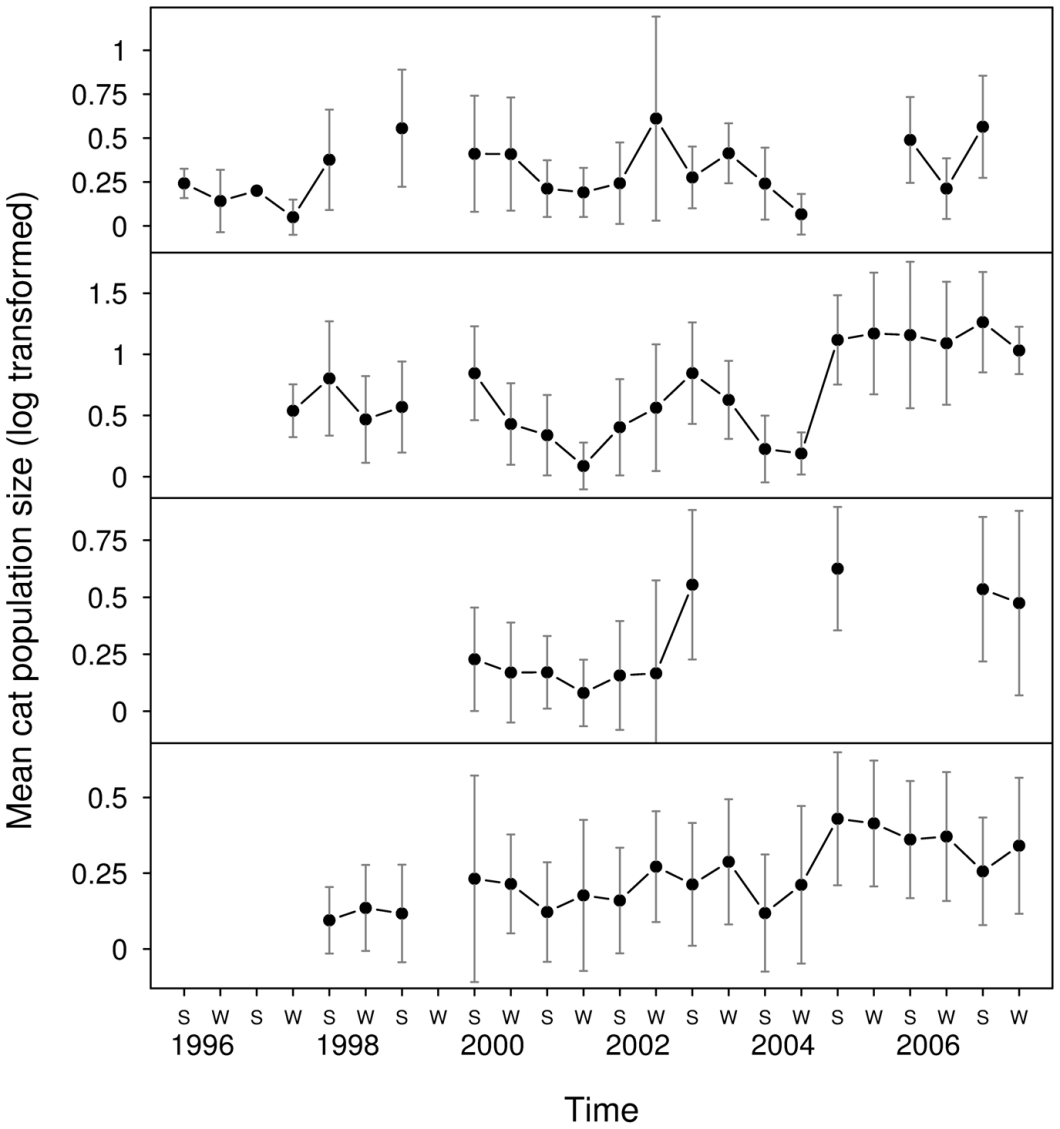
Averaged time variations in observed cat population size at the four study sites. The different panels show the time series for the four study sites (from top to bottom): Port-aux-Français, Port-Jeanne-d’Arc, Port-Couvreux and Ratmanoff. Points represent the mean of log-transformed cat population size estimates. The grey bars represent the respective standard deviation of the observed means on the logarithmic scale. The symbols ‘S’ and ‘W’ stand, respectively, for summer and winter.

Contrary to the fish data, the magnitude of temporal variations in cat abundance differed widely from site to site (see Fig. 2). Thus, we used the 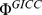
parametrization to quantify population synchrony among cat populations. More specifically, we considered the following state process:
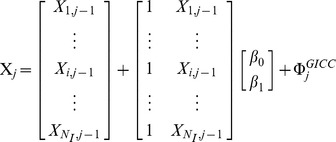
where 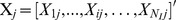 is the true (log) mean number of cats at site *i* in year *j*; 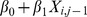 is the first order Gompertz model where 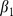 is a coefficient of density-dependence and 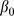 is a constant. Here 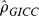 is an estimator of the strength of the average synchrony (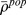) among cat populations (see Material and Methods section 1.3).

#### 2.3. Ethics Statement

The fieldwork has been made by qualified people according to the French legislation. Accreditation has been granted to the UMR-CNRS 5558 (accreditation number 692660703) for the feral cat program.

#### 2.4. Estimating spatial synchrony using the standard approach neglecting sampling variance: a ‘what if’ scenario analysis

Here we aimed to assess the bias of the average synchrony estimator 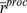 that we would have encountered if we had neglected sampling error associated with the fish and cat population size estimates. We considered two cases often encountered in synchrony studies. We first considered the case where sampling variance is completely neglected, i.e., when using only one population size estimate for each site and time step (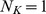). The second case we considered is when sampling variance is only partially accounted for, i.e., when abundance data are collected according to a replicated sampling protocol and the 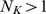 samples are aggregated into one estimate to reduce uncertainty about the population size estimate.

*Fish example*: based on the model parameter estimates, following the superpopulation model (see File S2 for details), we first generated a collection of *N_M_* sets of time series of ‘true’ population states 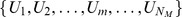. Second, within each *U_m_*, we averaged, for each site *i* and year *j, N_K_* estimates of (log) population size 
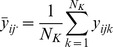
 drawn at random from a normal distribution: 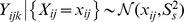. Since we aimed to explore the case where sampling variance is completely neglected, we considered one experimental fishing per year in our simulations. Third, we fitted a Gompertz model to each set of time series of averaged population size and quantified the synchrony strength by computing the average sample correlation (
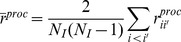
) within each set of time series of residuals of the Gompertz models.

*Cat example*: we used the same approach for the cat example except that within each *U_m_*, we averaged, for each site *i* and year *j, N_K_* log-transform estimates of population size 
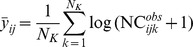
 drawn at random from a binomial distribution 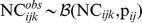 with 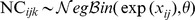. As for the simulation of fish abundance, we only considered one field session per site-time.

For both the fish and cat examples, we performed 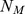 = 1000 simulations for different values of 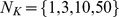. To assess the *ps*-bias of 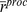, we compared the 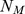 average correlations 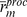 to the estimate obtained using the state-space modelling approach, that we considered as a gold standard of 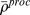 (see File S5).

## Results

### 1. Expected Bias of the Usual Synchrony Estimator in the Presence of Sampling Error

**Figure 3:**
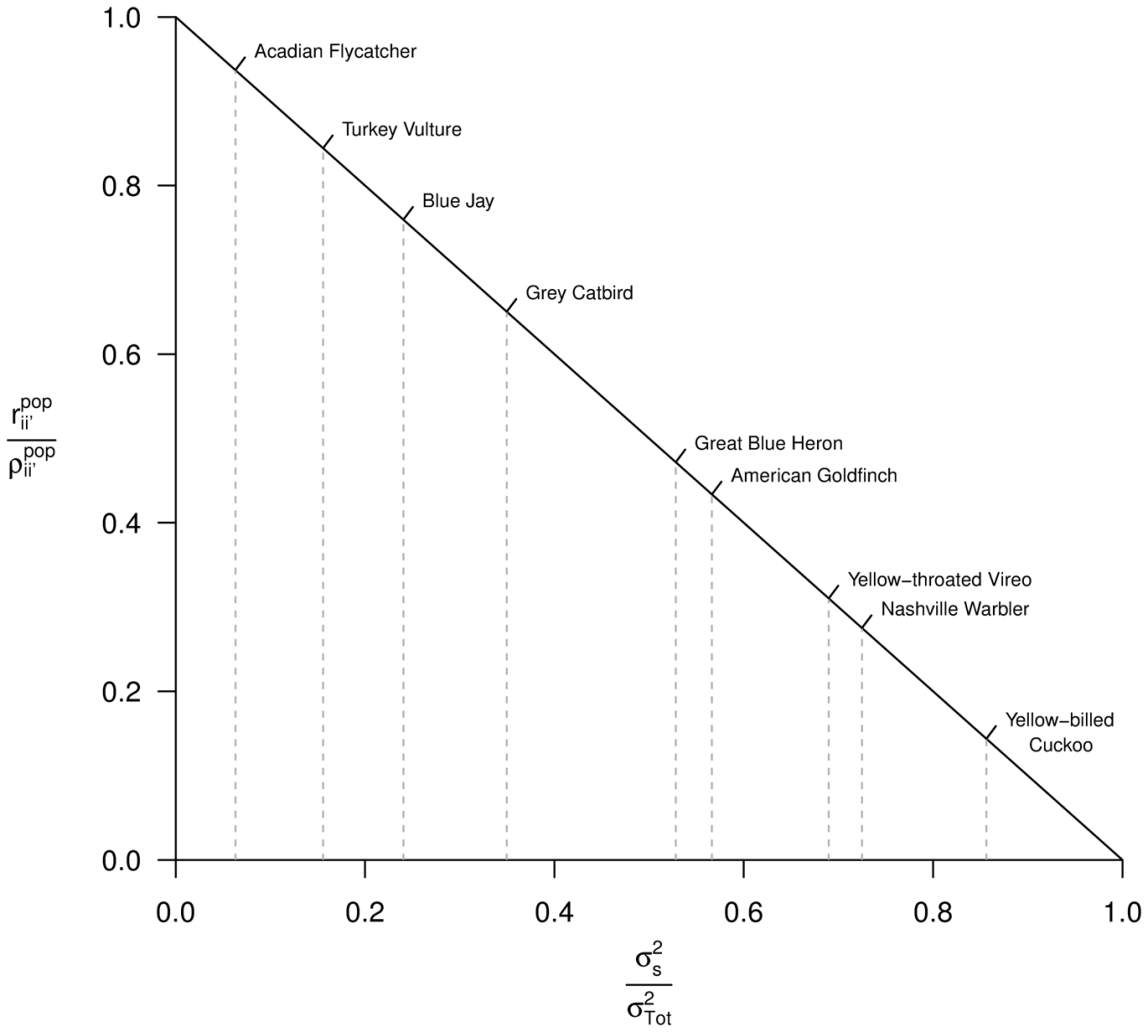
Bias of the cross-correlation estimator 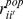 in presence of sampling error. The thick line represent the value of the ratio (bias) of the true correlation 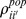 between two populations *i* and *i’* and of the Esperance 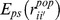 of the correlation estimator classically used to measure population synchrony among two time series of observed population size, in relation to the contribution of sampling variance 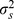 to the total temporal variance in observed population size 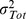. Dashed vertical bars represent the values of 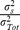
estimated by Link et al. [26] for 9 bird species.

Fig. 3 shows the extent of the bias of the usual synchrony estimator (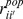) that can be encountered when sampling variance is neglected. For example, consider two perfectly synchronous populations (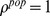) of Yellow-throated Vireo. Based on the estimates of the ratio between sampling and total variance reported by Link et al. [26], ignoring sampling variance would lead to an underestimation of the synchrony pattern of approximately 70% on average. More generally, Fig. 3 shows that a wide range of situations can be encountered: while ignoring sampling variance would lead to negligible bias for some species (e.g., Acadian Flycatcher), it can also, at least in theory, completely mask the synchrony pattern (e.g.,Yellow-billed Cuckoo).

### 2. Illustrative Examples

To fully specify our Bayesian models, we provided non-informative priors to all of the parameters. Specifically, we chose normal distribution with mean 0 and variance 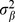 for 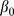 and 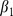, and uniform distribution on [0,1] for 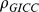. We chose inverse-gamma with both parameters equal to 0.001 for 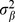,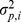, 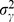, 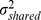 and 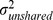, and exponential with inverse scale parameter equal to 1 for *θ*. All priors were selected as sufficiently vague in order to induce little prior knowledge.

For both state-space models, we generated three chains (MCMC) of length 1,200,000 and discarded the first 100,000 as burn-in. To accommodate memory constraints, we thinned the chains by taking all 100^th^ values. Convergence of the Markov chains was assessed using the Gelman and Rubin statistic (see [42] pp. 294–298).

#### 2.1. Estimation of spatial synchrony in fish and cat populations

Using our state-space models, we found a strong pattern of synchrony among fish populations (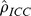: mean = 0.86, sd = 0.11, Table 1) as well as among cat populations (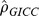: mean = 0.75, sd = 0.22, Table 2). Estimates of the density-dependence parameter 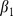 are mean(sd) −0.26(0.10) and −0.44(0.16) for the fish and the cat populations, respectively. The estimates of detection probabilities (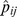 ) for the cat dataset range from 0.19 to 0.76. On each site the average detection probability are: 0.54 for Port-aux-Français, 0.61 for Port-Jeanne-d’Arc, 0.54 for Port-Couvreux and 0.52 for Ratmanoff. Estimates of 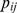 and their 95% credible intervals are available as supplementary file (File S6).

**Table 1:** Model parameter estimates for the fish example.

Parameters	Mean	SD	CI_95%_
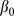	0.25	0.11	0.04;0.50
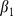	−0.26	0.10	−0.48; −0.08
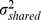	0.04	0.02	0.01;0.10
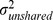	0.007	0.007	0.0006;0.0261
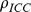	0.86	0.11	0.52;0.98
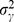	0.005	0.005	0.0004;0.020

Posterior mean, standard deviation and 95% credible interval of the model parameter estimates obtained for the state-space model fit on the fish dataset.

**Table 2:** Model parameter estimates for the cat example.

Parameters	Mean	SD	CI_95%_
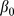	−1	0.38	−1.86; −0.33
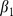	−0.44	0.16	−0.82; −0.15
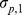	0.41	0.21	0.06;0.89
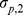	1.02	0.32	0.47;1.74
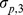	0.58	0.26	0.17;1.20
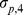	0.22	0.16	0.01∶0.62
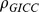	0.75	0.22	0.16;0.99
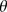	5.11	0.67	3.95;6.61
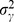	0.12	0.03	0.07;0.20

Posterior mean, standard deviation and 95% credible interval of the model parameter estimates obtained for the state-space model fit on the cat dataset. See the supplementary file (S6) for the estimates of 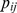.

#### 2.2. Estimating spatial synchrony using the standard approach neglecting sampling variance: a ‘what if’ scenario analysis

**Figure 4:**
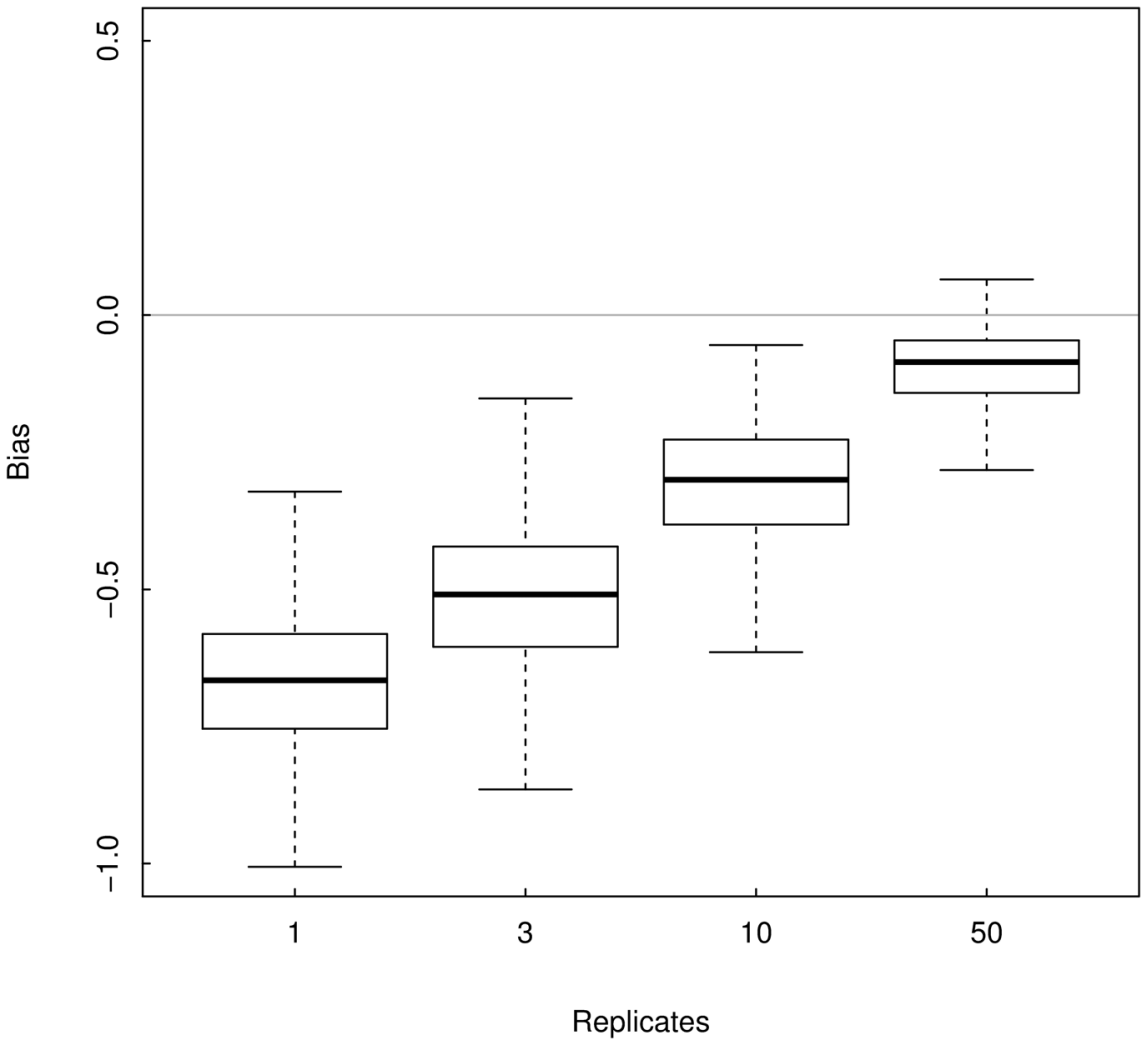
Monte Carlo estimates of the bias of the cross-correlation estimator 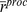 for the fish example. The boxplot represent the Monte Carlo distribution of cross-correlation estimates obtained when averaging 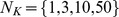 replicates of fish population size tainted by sampling error minus the synchrony estimate obtained using a state-space modelling approach accounting for sampling error i.e., a gold standard.

**Figure 5:**
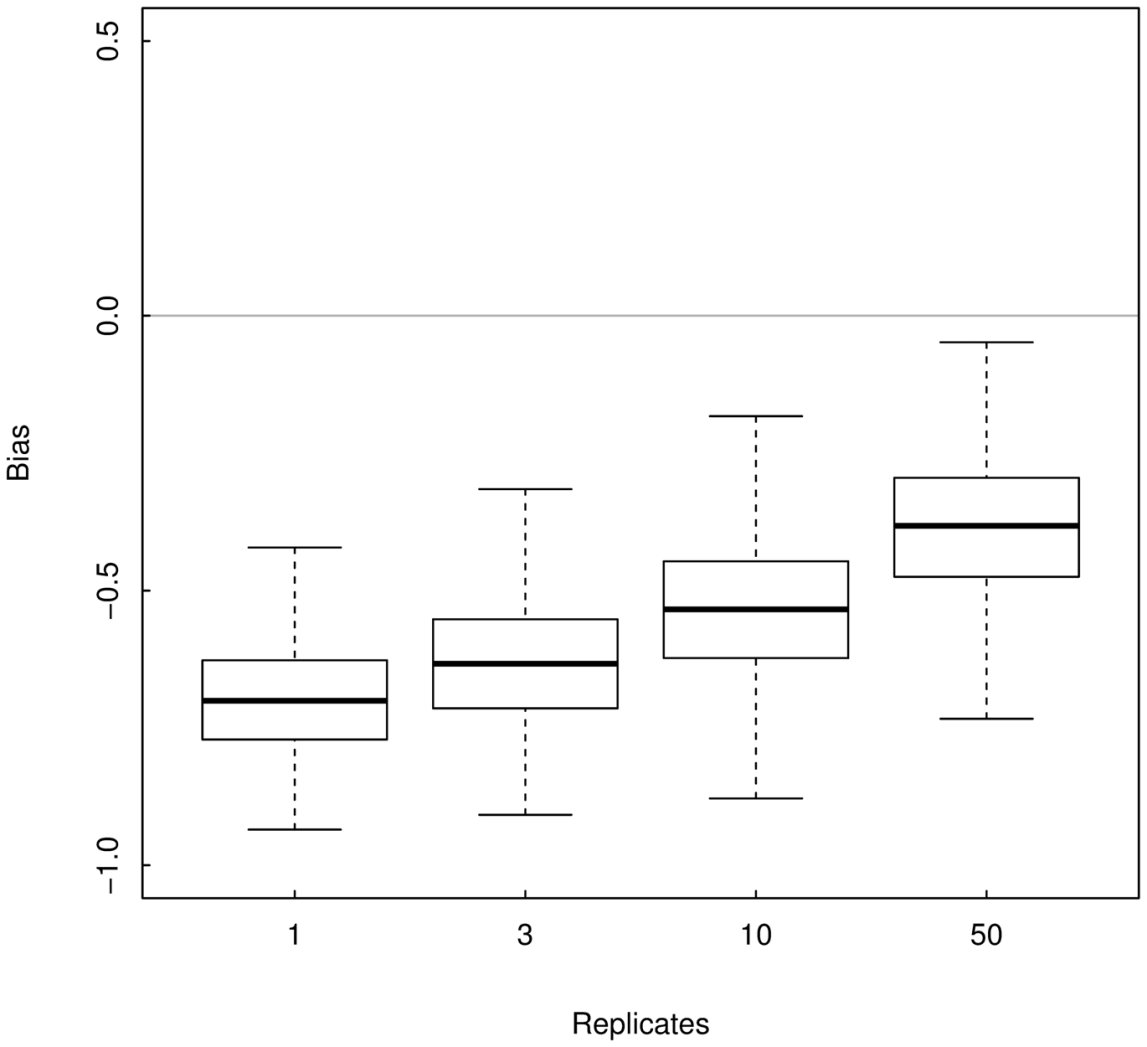
Monte Carlo estimates of the bias of the cross-correlation estimator 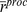 for the cat example. The boxplot represent the Monte Carlo distribution of cross-correlation estimates obtained when averaging 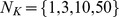 replicates of cat population size tainted by sampling error minus the synchrony estimate obtained using a state-space modelling approach accounting for sampling error i.e., a gold standard.

Monte Carlo simulations revealed that neglecting sampling variance (i.e., using 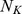 = 1 replicate of population size estimate), or partially accounting for sampling variance (i.e., using 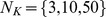 replicates of population size estimate) lead to underestimate the strength of spatial synchrony patterns (Figs. 4 and 5). For 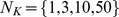, Monte Carlo estimates of average synchrony among fish populations are {mean (sd): 0.19 (0.12), 0.34 (0.13), 0.55 (0.11), 0.76 (0.08)}, *vs*. 0.86 (0.11) estimated by fitting the state-space model. Among cat populations we obtained {mean (sd): 0.06 (0.10), 0.12 (0.11), 0.22 (0.12), 0.37 (0.13)}, *vs*. 0.75 (0.22). As expected, the strength of density-dependence is overestimated: for fish populations the Monte Carlo estimates are {mean(sd): −0.91 (0.14), −0.82 (0.16), −0.73 (0.17), −0.67 (0.17)} *vs*. −0.26 (0.10) estimated by fitting the state-space model, and for cat populations we obtained {mean(sd): −0.90 (0.14), −0.80 (0.15), −0.68 (0.17), −0.62 (0.17)} *vs*. −0.44 (0.16).

## Discussion

### 1. Accounting for Sampling Error when Inferring Population Synchrony from Time-series Data

Using Bayesian state-space models in which process and sampling variances are separately defined, we showed that the temporal fluctuations in abundance of both fish and cat populations are strongly synchronised. As expected, the results of the Monte Carlo simulations based on the model parameter estimates show that ignoring sampling variance would not have enabled highlighting these patterns. When the ratio between sampling and total temporal variances is large, i.e., when we considered only one replicate of cat abundance per time unit, the synchrony estimates fall near zero. Thus, based on real data, we showed that neglecting sampling variance can completely mask a synchrony pattern whatever its ‘true’ strength. These results show that there is a clear need to account for sampling error to accurately quantify the strength of synchrony patterns. Assessing the bias of an estimator of the strength of the synchrony pattern in the presence of sampling error requires knowing the value of 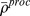, i.e., the superpopulation parameter describing the correlation among population processes. This was not possible for the two datasets analysed here because the true states of the populations were unknown. But for the purpose of this study, we considered synchrony estimates obtained with state-space models as true values (or gold standards) because such a modelling approach has negligible bias (File S5).

In this study we focus on the consequences of ignoring sampling variance on the estimation of spatial synchrony but other factors can modulate population synchrony estimates. For instance, in small populations, demographic stochasticity can tend to dominate environmental stochasticity and consequently population synchrony is expected to increase with population size [19], [43]. Ideally, the model fitted on the cat dataset should have included a demographic component in the process error. However, at present time there is not enough data for estimating demographic variance in the cat populations studied. We acknowledge that a fully realistic model should also have allowed for non-linear density dependence (e.g., theta-logistic model, [20]) and/or spatial variations in the strength of the density dependence. Moreover, given the life expectancy of both species studied here, age structure should have been taken into account [44]. In both illustrative examples, we assumed no age structure effect and a density-dependence linear and identical among populations. These assumptions were compromise between complexity and reality. Exploring the consequence of the misspecification of the form of the density-dependence on the quantification of synchrony among population processes was beyond the scope of this paper (e.g. [45]). Note however that if dispersal does not occur, theoretical works suggest that when non linearity or heterogeneity among populations are features of the population process then 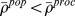 [29]. So the estimates 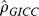 and 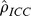
provided in this study for 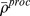
are likely to be lower bounds of the actual values, if it is assumed that dispersal between populations is null (fish data) or negligible (cat data). Non-linearity and heterogeneity among populations can easily be implemented in a Bayesian state-space model with the consequence that the hypothesis that 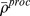 and 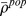
are equal will no more hold. In this case, 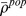
can be estimated by simulations or, in some cases, analytically, using parameter values of the model [29].

In this study, we showed that averaging replicates of population size estimates (or indices) allows decreasing the bias in the estimation of the strength of spatial synchrony. We also showed that the bias in the density-dependence estimation decreases as the number of replicates increases. However, we used up to 50 replicates in our simulations while in practice the sampling procedure rarely exceeds 3 replicates, mainly because of financial and logistic constraints. Such limitations (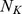 = 3) would have led to underestimating the strength of the synchrony pattern by 60% for fish populations and by 84% for cat populations. It follows that averaging few replicated samples does not guarantee that the synchrony strength will not be substantially underestimated. Thus, accurately quantifying the strength of spatial synchrony requires combining both a sampling protocol that enables the estimation of sampling variance and a statistical procedure allowing proper accounting for it.

In their study of the fish dataset, Tedesco et al. [24] used eqn 2 to correct the synchrony estimate *a posteriori*. But this approach has some limitations. For instance, they had to assume that sampling error is independent of population size and thus to select a fixed number of replicates within each time period; they worked with a balanced subset of the data (representing 73% of the data available) to cope with the homoscedasticity assumption. By accounting for sampling variance at the observation level, state-space models overcome these limitations. By doing so, all the available data are included in the analysis and all the model parameters are adjusted for sampling variance. Another appealing feature of the state-space model presented here is that it can easily accommodate for over-dispersion in count data.

### 2. What are the Likely Mechanisms Beyond Synchrony?

Altogether, our results suggest that ignoring sampling error, in addition to leading to spurious estimates of the strength of spatial synchrony among populations, increases the difficulty of identifying the mechanisms beyond synchrony. Three non-mutually exclusive factors are classically involved in driving synchrony: individual dispersal [4], [46], predation by a nomadic predator [2] and spatially correlated climatic conditions (Moran effect [3]). For example, to identify whether the Moran effect is acting, one can compare the correlation among climatic conditions to the correlation among time variations in population size. In case of climatically-driven population synchrony, these correlations are expected to be equal. But this holds only if (i) the biological assumptions of the Moran theorem apply (no dispersal between populations and identical linear density-dependence structures), and (ii) both population sizes and climatic variables are known without error. In practice, both population sizes and climatic variables are tainted by sampling errors. Consequently, correlation estimators neglecting sampling variance will be downward biased estimators of the theoretical correlations. For some climatic variables the sampling error impact may be negligible but this should be assessed case by case. Whenever the contribution of the sampling variance to the total temporal variance has a strong impact on the correlation estimator, it should be accounted for.

Disentangling synchronisation mechanisms from patterns is challenging, especially when sampling error is neglected. However, estimates of synchrony strength may provide clues regarding the most plausible mechanisms.

The synchrony parameter among the fish populations is estimated to 0.86, i.e., very close to the correlation of 0.87 reported by Tedesco et al. [24] among the annual discharge index of the corresponding basins. It was not possible to account for sampling variance when estimating the correlation among annual discharge index (data not available) and thus this estimate is likely to be an underestimation of the true value. In spite of this, this result is consistent with the probable role of hydrological conditions on the dynamics of the fish populations and suggests that the Moran effect is acting. This is reinforced by the fact that the populations studied are living in different river systems, i.e., disconnected populations, excluding dispersal as a synchronising agent (see [24] for more details). Such a demonstration of a Moran effect would not have been possible by considering the synchrony estimates of 0.21 obtained when sampling error is neglected.

For the cat example, considering (i) the absence of cat predator on the Kerguelen archipelago and (ii) the strong genetic structure among the cat populations (suggesting low dispersal among populations [47]), and (iii) the strong strength of synchrony (0.75) observed, climate appears to be the most plausible mechanism. A possible scenario is that the cat population dynamic is strongly related to the population dynamics of its main prey, the rabbit. The rabbit (*Oryctolagus cuniculus*) population dynamics is likely under the influence of the yearly plant biomass production which is itself influenced by climatic conditions. This hypothesis is reinforced given that (i) climatic conditions have been shown to be synchronous at a level higher than 0.5 at a spatial scale similar to our study [48] and (ii) the yearly production of plant biomass is also synchronous (0.70) among the four study sites [40], [49]. Here, the yearly plant biomass production was estimated from the Normalized Difference Vegetation Index and is tainted by sampling error [49]. In absence of estimate of sampling variance in yearly production of biomass it was not possible to obtain a synchrony estimate corrected for it, but it is likely that the true value is much higher than 0.7. The synchrony of 0.75 between cat populations can be viewed as a result of the synchrony in yearly vegetation production, mediated by rabbit population dynamics. Since dispersal cannot be excluded by design in this system, further studies are needed to assess the relative contribution of climate and dispersal to the synchronization of cat populations. Dispersal and climate are likely to act on local population dynamics at different spatial scales. A strategy for disentangling their contribution to the synchrony pattern would consist in exploring how fast the correlation among populations decrease with geographic distance, i.e., in quantifying the ‘spatial scaling of population synchrony’ [50], [51]. Assuming that a larger number of study sites than considered in this study was monitored, the spatial scaling of population synchrony can be assessed by computing all the pair correlations among the 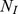 time series of estimates of residual process variations (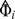) and then comparing these estimates to the corresponding inter-site distances. Alternatively, it could be possible to extend the state space modeling approach presented here to include a spatial covariance modeled as a function of the inter-site distances (e.g., [52]).

### 3. Conclusion

We showed that when the contribution of sampling variance to the total temporal variance is set to values typical for natural populations, (i) ignoring sampling variance can mask a synchrony pattern, and (ii) averaging few replicates of population size estimates poorly performed in decreasing the bias of the estimator of the synchrony strength. Bayesian state-space models, as the one presented in this study, provide a flexible way of quantifying the strength of synchrony patterns from most population size data encountered in field studies, including over-dispersed count data. We strongly encourage further studies aiming at quantifying the strength of population synchrony to account for uncertainty in population size estimates.

## Supporting Information

File S1 **List of the main mathematical notations.**
(DOC)

File S2 **Superpopulation model definition and notation.**
(DOC)

File S3
**Definition of** 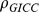 **and** 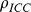 **.**
(DOC)

File S4 **R-program to fit the state space model and the fish dataset.**
(R) Click here for additional data file.

File S5 **Monte Carlo assessment of the bias of the state-space model approach presented in this study to quantify the strength of the spatial synchrony among populations from population size data tainted by sampling error.**
(DOC)

File S6
**Detection probability estimates (** 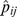 **) obtained for the state-space model fitted on the cat dataset.**
(DOC)
