# Supplementary material for: Accounting for Sampling Error When Inferring Population Synchrony from Time-Series Data: A Bayesian State-Space Modelling Approach with Applications
Source: PLoS One. 2014 Jan 29;9(1):e87084. doi: 10.1371/journal.pone.0087084 (PMC3906118; doi:10.1371/journal.pone.0087084)
Supplement: File S1 — List of the main mathematical notations. (DOC) [file pone.0087084.s001.doc]

**File S1. List of the main mathematical notations.**

$r_{ii'}^{pop}$ Estimator of the population correlation, i.e., the zero-lag correlation computed between two time series *i* and *i*’ of observed (log) population sizes.

$r_{ii'}^{proc}$ Estimator of the process error correlation, i.e., the zero-lag correlation computed among residuals of population dynamic models fitted on two time series *i* and *i*’ of observed (log) population sizes.

$N_{I},N_{J},N_{K}$ Numbers of sites, repetitions (number of realisations of the population process through time) and replicates (number of realisations of the sampling process), respectively.

$x_{ij}$ Realisation of the random variable $X_{ij}$ modelling the unobservable true (log) population size at site *i* at time *j*.

$\beta_{1},\beta_{2},...,\beta_{h}$ Parameters defining the strength of the density-dependence up to order *h*.

$z_{ij}$ Realisation of the random variable $Z_{ij}$ modelling the process error at site *i* at time *j*, i.e., time variations in true (log) population size not accounted for by density-dependence.

$\sigma_{p,i}^{2},\sigma_{p,ii'}$ Variance of the residual process fluctuations at site *i* and covariance of the residual process fluctuations between two sites *i* and *i*’, respectively.

$E_{p}\left(\cdot\right)$ *p*-expectation, i.e., the expectation under the population process.

$V_{p}\left(\cdot\right)$ *p*-variance, i.e., the variance under the population process.

$C_{p}\left(\cdot,\cdot\right)$ *p*-covariance, i.e., the covariance under the population process.

*U* Particular set of $N_{I}$ time series of $N_{J}$ true (log) population sizes.

$y_{ij}$ Realisation of the random variable $Y_{ij}$ modelling the observed (log) population size at site *i* at time *j*.

$o_{ijk}$ Realisation of the random variable $O_{ijk}$modelling the sampling error at site *i* at time *j* for replicate *k.*

$\sigma_{s,ij}^{2}$ Sampling variance for site *i* at time *j*.

$E_{s}\left(\cdot\right)$ *s*-expectation, i.e., expectation under the sampling process.

$V_{s}\left(\cdot\right)$ *s*-variance, i.e., the variance under the sampling process.

$C_{s}\left(\cdot,\cdot\right)$ *s*-covariance, i.e., the covariance under the sampling process.

*s* Particular set of $N_{I}N_{K}$ time series of observed (log) population sizes.

$\rho_{U,ii'}^{pop},\rho_{U,ii'}^{proc}$ Finite population parameters for *U*, defining the correlation between two time series *i* and *i*’ of true (log) population sizes and process errors, respectively.

$\rho_{ii'}^{pop},\rho_{ii'}^{proc}$ Superpopulation parameters defining the population correlation and the process error correlation between two sites *i* and *i*’, respectively.

$\bar{\rho}^{pop},\bar{\rho}^{proc}$ Average population synchrony and average synchrony among process errors, respectively.

$\sigma_{Tot,i}^{2}$ Total temporal variance in observed (log) population sizes at site *i* (magnitude of the temporal variations due to both process and sampling variations).

$S_{Tot,i}^{2},S_{Tot,ii'}$ Estimators of the total temporal variance in observed (log) population sizes at site *i* and of the total temporal covariance in observed (log) population size between two sites *i* and *i*’.

$S_{s}^{2}$ Estimator of the sampling variance.

$f(\cdot)$ Function of past population states representing deterministic variations in local population dynamics.

$\hat{\rho}_{ICC},\hat{\rho}_{GICC}$ Intra-Class correlation and Generalised Intra-Class Correlation estimators, which both estimate $\bar{\rho}^{proc}$.

$\tau_{j}$ *j*^th^ realisation of the random variable $\tau$ (random effect) modelling temporal variations in residual process variations shared among sites.

$\eta_{ij}$ *j*^th^ realisation of the random variable $\eta$ at site *i* (random effect) modelling temporal variations in residual process variations unshared among sites.

$\hat{\sigma}_{shared}^{2}$,$\hat{\sigma}_{unshared}^{2}$Estimators of the variances of the temporal fluctuations in residual process errors that are shared and unshared among sites, respectively.

$E_{ps}\left(\cdot\right)$ *ps*-expectation, which is the expectation under both the population and the sampling processes.
