# Supplementary material for: Accounting for Sampling Error When Inferring Population Synchrony from Time-Series Data: A Bayesian State-Space Modelling Approach with Applications
Source: PLoS One. 2014 Jan 29;9(1):e87084. doi: 10.1371/journal.pone.0087084 (PMC3906118; doi:10.1371/journal.pone.0087084)
Supplement: File S2 — Superpopulation model definition and notation. (DOC) [file pone.0087084.s002.doc]

**File S2. Superpopulation model definition and notation**

**1. Superpopulation model and notation**

Exploring the size of the bias of the approach classically used for quantifying population synchrony from time series of observed log population size requires that particular consideration is given to the generating process of such data. To begin, we specify a superpopulation model that mimics the main mechanisms involved in generating time series of observed log population size. This model may be used to generate pseudo-data and provides the foundations of our inferential framework, but it must not be confounded with the model used to estimate the population synchrony, which is introduced in section 1.3 of the main text. Note that the term ‘superpopulation’ is more widely used in Statistics (especially in Survey Sampling Theory) than in Ecology, but see , page 6 or for applications in this field. Note also that a different meaning of the term ‘superpopulation’ may also be encountered in statistical Ecology (e.g., ) where ‘*a 'superpopulation' is defined as the total number of animals that were alive and available to be captured during at least one sampling period of the study and is thus composed of the recruits to the population across all sampling periods*’ . The two meanings are easily distinguishable according to the context.

For the sake of simplicity, we consider here populations synchronised by a Moran effect. More specifically, we considered populations subject to the same linear density-dependence and not connected by dispersal. The model is able to reproduce (i) the temporal variability in true log population size resulting from the action of both intrinsic (density-dependence) and extrinsic factors (e.g*.*, correlated climatic variations), and (ii) the variability resulting from sampling error (see File S1 for a list of the main mathematical notations used).

Let $\text{x}_{j}$ be a *N_I_-*element vector $\left[x_{1j},\ldots,x_{ij},...,x_{N_{I}j}\right]^{'}$of true log population sizes on site $i\in I=\{1,2,...,N_{I}\}$ at time $j\in J=\{1,2,...,N_{J}\}$, where *I* and *J* are two ordered sets of indices, with *j* typically indexes years. $\text{x}_{j}$can be viewed as a particular realisation of a multivariate Gompertz process (thereafter called the population process):

$$\text{X}_{j} = \left[\begin{matrix} X_{1,j-1} \\ \vdots\\ X_{i,j-1} \\ \vdots\\ X_{N_{I},j-1} \end{matrix}\right]+\left[\begin{matrix} 1 & X_{1,j-1} & \ldots& X_{1,j-h} \\ \vdots& \vdots& \ddots& \vdots\\ 1 & X_{i,j-1} & \ldots& X_{i,j-h} \\ \vdots& \vdots& \ddots& \vdots\\ 1 & X_{N_{I},j-1} & \ldots& X_{N_{I},j-h} \end{matrix}\right]\left[\begin{matrix} \beta_{0} \\ \beta_{1} \\ \vdots\\ \vdots\\ \beta_{h} \end{matrix}\right]+\text{Z}$$

where $\text{X}_{j}$ is a *N_I_-*element vector of random variables $\left(X_{ij}\right)$,$\beta_{0}$is an intercept and $\{\beta_{1},...,\beta_{h}\}$ representing the strength of density-dependence up to order *h*. $\text{Z}$ is an *N_I_-*element vector of random variables *Z_i_* generating $N_{I}$ process errors *z_ij_* at time *j* that are variations in true log population size not accounted for by density-dependence. These random variables are assumed to have a certain joint probability distribution characterized by the first and second-order moments:

$$E_{p}\left(Z_{i}\right)=0$$

$$C_{p}\left(Z_{i},Z_{i'}\right)=\Sigma$$

with $\Sigma=\left[\begin{matrix} \sigma_{p,1}^{2} & \sigma_{p,12} & \cdots& \sigma_{p,1i} & \cdots& \sigma_{p,1N_{I}} \\ \sigma_{p,21} & \sigma_{p,2}^{2} & \cdots& \sigma_{p,2i} & \cdots& \sigma_{p,2N_{I}} \\ \vdots& \vdots& \ddots& \vdots& & \vdots\\ \sigma_{p,i1} & \sigma_{p,i2} & \cdots& \sigma_{p,i}^{2} & \cdots& \sigma_{p,iN_{I}} \\ \vdots& \vdots& & \vdots& \ddots& \vdots\\ \sigma_{p,N_{I}1} & \sigma_{p,N_{I}2} & \cdots& \sigma_{p,N_{I}i} & \cdots& \sigma_{p,N_{I}}^{2} \end{matrix}\right]$

where $\sigma_{p,i}^{2}$ is the residual process variance of site *i* (i.e., the magnitude of stochastic variations on site *i*) and $\sigma_{p,ii'}$is the covariance between residual process variations among two sites *i* and *i’*. We used the subscript ‘*p*’ for the notation of the operator of expectations $E_{p}(\cdot)$and covariance $C_{p}(\cdot)$to recall that the source of stochasticity involved in their definition is the population process. We call ‘repetitions’ the $N_{J}$ realisations of $\text{X}_{j}$ required to generate a set (denoted as $U=\left\{\text{x}_{j},j\in J\right\}$) of $N_{I}$ time series of $N_{J}$ true log population size. In real world, there is only one fixed *U* corresponding to a given set of sites and a given time period. However, for inference purpose we need to consider the infinite collection of *U,* noted $U_{1},U_{2},\ldots,U_{m},...,$ which can be generated from the population process.

In the $N_{I}$ sites, $\text{x}_{j}$is not directly observable and the observers record — an estimate of $\text{x}_{j}$tainted by sampling error —

$\text{y}_{j}=\left[y_{1j1},...,y_{1jk},...,y_{1jN_{K}},...,y_{ij1},...,y_{ijk},...,y_{ijN_{K}},...,y_{N_{I}j1},...,y_{N_{I}jk},...,y_{N_{I}jN_{K}}\right]^{'}$with $y_{ijk}$($k\in K=\{1,2,...,N_{K}\}$) denoting the (log) number of individuals counted on the *k^th^* sampling unit at site *i* and time *j*. Note that for the sake of simplicity we consider here that the number of sampling units did not change according to site and time*.* $\text{y}_{j}$can be viewed as a realisation of the following sampling process:

$$\text{Y}_{j}\left|\left\{\mathbf{X}_{j}=\mathbf{x}_{j}\right\}\right.=\text{D}_{j}\mathbf{x}_{j}+\text{ O}_{j}$$

where$\text{Y}_{j}$ is an $N_{I}N_{K}$*-*element vector of random variables $\left(Y_{ijk}\right)$; $\text{D}_{j}$ is an $N_{I}N_{K}\times N_{I}$ matrix of 0s and 1s that translates the $N_{I}$ true log population size at time *j* into $N_{I}N_{K}$ true log population sizes at time *j*; $\text{O}_{j}$ is an $N_{I}N_{K}$*-*element vector of random variables $\left(O_{ijk}\right)$ generating sampling errors and assumed to have a certain joint probability distribution characterized by the first and second-order moments:

$$E_{s}\left(O_{ijk}\right)=0$$

$$C_{s}\left(O_{ijk},O_{i'j'k'}\right)=\left\{\begin{matrix} \sigma_{s,ij}^{2} if i=i', j=j', k=k' \\ 0 else \end{matrix}\right.$$

where $\sigma_{s,ij}^{2}$ is the common sampling variance for the *N_K_* sampling units for the site *i* at time *j*. In the specification of *C_s_* note that there is no covariance between sampling units, whatever site or time. We used the subscript ‘*s*’ for the notation of the operator of expectations $E_{s}(\cdot)$and covariance $C_{s}(\cdot)$to recall that the source of stochasticity involved in their definitions is the sampling process. We call ‘replicates’ the $N_{K}$ realisations of $\text{Y}_{j}$ required to generate a set (noted *s*) of $N_{I}N_{K}$ time series of observed log population sizes. In real world, there is only one fixed *s* resulting from observing a fixed *U*. However, for inference purpose we need to consider the infinite collection of *s* (given *U*)*,* noted $s_{1},s_{2},\ldots,s_{n},...$ that can be generated from the sampling process.

**2. Notions of population synchrony**

Let $\rho_{ii',U}^{pop}$ be the finite population parameter for the set *U* (denoted in subscript) defining the correlation among two time series *i* and *i*’ of true log population sizes. According to the superpopulation model defined above, $\rho_{ii',U}^{pop}$ is a *p*-unbiased estimator of the correlation $\rho_{ii'}^{pop}$ defining the theoretical synchrony between the two populations *i* and *i*’. It follows that the theoretical average population synchrony $\bar{\rho}^{pop}$ is given by the average of all pairwise correlations among the *N_I_* populations: $\bar{\rho}^{pop}=\frac{2}{N_{I}(N_{I}-1)}\sum_{i<i'} \rho_{ii'}^{pop}$. Since we considered populations that are (i) synchronised by a Moran effect, and (ii) subject to the same linear density-dependence and not connected by dispersal, the theoretical synchrony between two populations *i* and *i’* can also be directly derived from the correlation among their process errors $\left(\rho_{ii'}^{proc}=\frac{\sigma_{p,ii'}}{\sigma_{p,i}\sigma_{p,i'}}\right)$. Indeed, under these conditions, the Moran theorem applies and provides$\rho_{ii'}^{pop}=\rho_{ii'}^{proc}$. Note that when density-dependence is non-linear and/or is heterogeneous between populations, this equality no longer holds and$\rho_{ii'}^{pop}$ becomes a function of the synchrony among population processes and of the parameters describing heterogeneous (and/or non-linear) density-dependence between the populations *i* and *i*’ . Generally, this leads to$\rho_{ii'}^{pop}<\rho_{ii'}^{proc}$. In this situation, the correlation among climatic conditions is expected to be equal to the correlation among population processes ($\rho_{ii'}^{proc}$) only.

**References**

1. Eberhardt LL, Thomas JM (1991) Designing Environmental Field Studies. Ecological Monographs 61: 53-73.

2. Legendre P, Legendre L (1998) Numerical Ecology. Amsterdam, The Netherlands: Elsevier. 853 p.

3. Aubry P, Debouzie D (2000) Geostatistical estimation variance for the spatial mean in two-dimensional systematic sampling. Ecology 81: 543-553.

4. Aubry P, Debouzie D (2001) Estimation of the mean from a two-dimensional sample: the geostatistical model-based approach. Ecology 82: 1484-1494.

5. Carroll EL, Childerhouse SJ, Fewster RM, Patenaude NJ, Steel D, et al. (2013) Accounting for female reproductive cycles in a superpopulation capture–recapture framework. Ecological Applications 23: 1677-1690.

6. Wen Z, Pollock K, Nichols J, Waser P (2011) Augmenting superpopulation capture–recapture models with population assignment data. Biometrics 67: 691-700.

7. Langton SL, Aebischer NA, Robertson PR (2002) The estimation of density dependence using census data from several sites. Oecologia 133: 466-473.

8. Royama T (1992) Analytical population dynamics. London: Chapman & Hall.

9. Hugueny B (2006) Spatial synchrony in population fluctuations: extending the Moran theorem to cope with spatially heterogeneous dynamics. Oikos 115: 3-14.

10. Royama T (2005) Moran effect on nonlinear population processes. Ecological Monographs 75: 277-293.
