# Supplementary material for: Accounting for Sampling Error When Inferring Population Synchrony from Time-Series Data: A Bayesian State-Space Modelling Approach with Applications
Source: PLoS One. 2014 Jan 29;9(1):e87084. doi: 10.1371/journal.pone.0087084 (PMC3906118; doi:10.1371/journal.pone.0087084)
Supplement: File S3 — Definition of and . (DOC) [file pone.0087084.s003.doc]

**File S3. Definition of $\rho_{GICC}$and** $\rho_{ICC}$

**1. Definition of** $\rho_{GICC}$

In this section we aimed at demonstrating that the Generalised Intra-Class Correlation $\rho_{GICC}$ measures the average correlation between times series of process errors$\bar{\rho}^{proc}$. Without loss of generality we considered $N_{I}=2$ sites. Consider two time series of process errors $\text{z}_{1}=\{z_{1j},j\in J\}$ and $\text{z}_{2}=\{z_{2j},j\in J\}$) generated according to:

$\left[\begin{matrix} Z_{1} \\ Z_{2} \end{matrix}\right]=\left[\begin{matrix} \sigma_{p,1}\left(\sqrt{\rho_{GICC}}\cdot\tau+\sqrt{1-\rho_{GICC}}\cdot\eta_{1}\right) \\ \sigma_{p,2}\left(\sqrt{\rho_{GICC}}\cdot\tau+\sqrt{1-\rho_{GICC}}\cdot\eta_{2}\right) \end{matrix}\right]$ eqn a

where is the residual process variance of site $i=1,2$; $\tau$ is a random variable generating the random term at time *j*, which represents shared variations in process errors among sites; $\eta_{i}$($i=1,2$) is a random variable generating random terms at time *j*, which represents unshared variations in process errors among sites.

By definition we have:

$$\bar{\rho}^{proc}=\frac{C_{p}(Z_{1},Z_{2})}{\sigma_{p,1}\sigma_{p,2}}$$

Given that $C_{p}(Z_{1},Z_{2})=E_{p}[(Z_{1}-E_{p}(Z_{1}))(Z_{2}-E_{p}(Z_{2}))]$ and that $E_{p}(Z_{1})=E_{p}(Z_{2})=0$, we have:

$$\bar{\rho}^{proc}=\frac{E_{p}(Z_{1}Z_{2})}{\sigma_{p,1}\sigma_{p,2}}$$

and substituting (eqn a) for the $Z_{1}$ and $Z_{2}$ it follows that:

$$\begin{matrix} \bar{\rho}^{proc}=\frac{\sigma_{p,1}\sigma_{p,2}E_{p}\left[\left(\sqrt{\rho_{GICC}}\cdot\tau+\sqrt{1-\rho_{GICC}}\cdot\eta_{1}\right)\left(\sqrt{\rho_{GICC}}\cdot\tau+\sqrt{1-\rho_{GICC}}\cdot\eta_{2}\right)\right]}{\sigma_{p,1}\sigma_{p,2}} \\ =E_{p}\left[\left(\sqrt{\rho_{GICC}}\cdot\tau+\sqrt{1-\rho_{GICC}}\cdot\eta_{1}\right)\left(\sqrt{\rho_{GICC}}\cdot\tau+\sqrt{1-\rho_{GICC}}\cdot\eta_{2}\right)\right] \\ =E_{p}\left[\rho_{GICC}\cdot\tau^{2}+\sqrt{\rho_{GICC}}\cdot\sqrt{1-\rho_{GICC}}\cdot\tau\eta_{1}+\sqrt{\rho_{GICC}}\cdot\sqrt{1-\rho_{GICC}}\cdot\tau\eta_{2}+(1-\rho_{GICC})\cdot\eta_{1}\eta_{2}\right] \\ =\rho_{GICC}\cdot E_{p}(\tau^{2})+\sqrt{\rho_{GICC}}\cdot\sqrt{1-\rho_{GICC}}\cdot E_{p}(\tau\eta_{1}) \\ +\sqrt{\rho_{GICC}}\cdot\sqrt{1-\rho_{GICC}}\cdot E_{p}(\tau\eta_{2})+(1-\rho_{GICC})\cdot E_{p}(\eta_{1}\eta_{2}) \end{matrix}$$

Since the $\tau$, $\eta_{1}$ and $\eta_{2}$are mutually independent with zero means and unit variances, it follows that $E_{p}(\tau\eta_{1})=E_{p}(\tau\eta_{2})=E_{p}(\eta_{1}\eta_{2})=0$ and that$E_{p}(\tau^{2})=\sigma_{\tau}^{2}=1$, leading to $\rho_{GICC}=\bar{\rho}^{proc}$.

**2. Definition of** $\rho_{ICC}$

In this section we aimed at demonstrating that when the magnitude of residuals process variations are similar among sites — as such we can consider $\sigma_{p,i}^{2}=\sigma_{p}^{2} \forall i$— the Intra-Class Correlation $\rho_{ICC}$ measures the average correlation between times series of process errors $\bar{\rho}^{proc}$. Without loss of generality we considered $N_{I}=2$ sites. Consider two time series of process errors $\text{z}_{1}=\{z_{1j},j\in J\}$ and $\text{z}_{2}=\{z_{2j},j\in J\}$) generated according to:

$\left[\begin{matrix} Z_{1} \\ Z_{2} \end{matrix}\right]=\left[\begin{matrix} \tau\cdot\sigma_{shared}+\eta_{1}\cdot\sigma_{unshared} \\ \tau\cdot\sigma_{shared}+\eta_{2}\cdot\sigma_{unshared} \end{matrix}\right]$ eqn b

with $\sigma_{p}^{2} =\sigma_{shared}^{2}+\sigma_{unshared}^{2}$. In (eqn. b), $\sigma_{shared}^{2}$and $\sigma_{unshared}^{2}$ are the variances of the residuals

process variations shared and unshared among sites, respectively; $\tau$ is a random variable generating the random term at time *j*, which represents shared variations in process errors among sites; $\eta_{i}$($i=1,2$) is a random variable generating random terms at time *j*, which represents unshared variations in process errors among sites.

By definition we have:

$$\bar{\rho}^{proc}=\frac{C_{p}(Z_{1},Z_{2})}{\sigma_{p,1}\sigma_{p,2}}=\frac{C_{p}(Z_{1},Z_{2})}{\sigma_{p}^{2}}=\frac{C_{p}(Z_{1},Z_{2})}{\sigma_{shared}^{2}+\sigma_{unshared}^{2}}$$

Given that $C_{p}(Z_{1},Z_{2})=E_{p}[(Z_{1}-E_{p}(Z_{1}))(Z_{2}-E_{p}(Z_{2}))]$ and that $E_{p}(Z_{1})=E_{p}(Z_{2})=0$, we have:

$$\bar{\rho}^{proc}=\frac{E_{p}(Z_{1}Z_{2})}{\sigma_{shared}^{2}+\sigma_{unshared}^{2}}$$

and substituting (eqn b) for the $Z_{1}$ and $Z_{2}$ it follows that:

$$\begin{matrix} \bar{\rho}^{proc}=\frac{E_{p}\left[\left(\tau\cdot\sigma_{shared}+\eta_{1}\cdot\sigma_{unshared}\right)\left(\tau\cdot\sigma_{shared}+\eta_{2}\cdot\sigma_{unshared}\right)\right]}{\sigma_{shared}^{2}+\sigma_{unshared}^{2}} \\ =\frac{E_{p}\left[\tau^{2}\cdot\sigma_{shared}^{2}+\tau\cdot\eta_{2}\cdot\sigma_{shared}\cdot\sigma_{unshared}+\tau\cdot\eta_{1}\cdot\sigma_{shared}\cdot\sigma_{unshared}+\eta_{1}\cdot\eta_{2}\cdot\sigma_{unshared}^{2}\right]}{\sigma_{shared}^{2}+\sigma_{unshared}^{2}} \\ =\frac{E_{p}(\tau^{2})\cdot\sigma_{shared}^{2}+\sigma_{shared}\sigma_{unshared}\cdot\left[E_{p}(\tau\cdot\eta_{2})+E_{p}(\tau\cdot\eta_{1})\right]+E_{p}(\eta_{1}\cdot\eta_{2})\cdot\sigma_{unshared}^{2}}{\sigma_{shared}^{2}+\sigma_{unshared}^{2}} \end{matrix}$$

Since the $\tau$, $\eta_{1}$ and $\eta_{2}$are mutually independent with zero means and unit variances, it follows that $E_{p}(\tau\cdot\eta_{1})=E_{p}(\tau\cdot\eta_{2})=E_{p}(\eta_{1}\cdot\eta_{2})=0$ and that$E_{p}(\tau^{2})=1$, leading to,

$$\bar{\rho}^{proc}=\frac{\sigma_{shared}^{2}}{\sigma_{shared}^{2}+\sigma_{unshared}^{2}}$$
