# Supplementary material for: Accounting for Sampling Error When Inferring Population Synchrony from Time-Series Data: A Bayesian State-Space Modelling Approach with Applications
Source: PLoS One. 2014 Jan 29;9(1):e87084. doi: 10.1371/journal.pone.0087084 (PMC3906118; doi:10.1371/journal.pone.0087084)
Supplement: File S5 — Monte Carlo assessment of the bias of the state-space model approach presented in this study to quantify the strength of the spatial synchrony among populations from population size data tainted by sampling error. (DOC) [file pone.0087084.s005.doc]

**File S5. Monte Carlo assessment of the bias of the state-space model approach presented in this study to quantify the strength of the spatial synchrony among populations from population size data tainted by sampling error.**

We performed numerical simulations to assess the bias of the state-space model approach presented in this study to quantify the strength of the spatial synchrony among populations based on (log) population size data tainted by sampling error. Since a previous study have already shown that such a modelling approach enables to obtain near unbiased estimate of process and sampling variances , we skipped the density-dependence estimation phase and we based our simulations directly on time series of process errors tainted by sampling error. Moreover, without loss of generality, we considered normally distributed sampling errors. We proceeded in three steps. Firstly, we generated a set (*U*) of $N_{I}$=20 time series of $N_{J}$=50 process errors ($z_{ij}$). Specifically, within each *U,* process errors were drawn at random from a multivariate normal distribution:

$\text{Z}_{j}\sim\mathrm{MVN}\left(0,\Sigma\right)$ eqn 1

where $0$is a $N_{I}$-element vector of zeros and ∑ is a $N_{I}\times N_{I}$ variance-covariance matrix with all diagonal elements equal to $\sigma_{p,ii}^{2}=1$ (the process error variance) and all off-diagonal elements equal to $\bar{\rho}^{proc}$ (the average synchrony among population processes).

Secondly, we generated a set (*s*) of $N_{I}N_{K}$ time series of process errors tainted by sampling errors according to the following sampling process:

$$\text{Y}_{j}|\text{\{Z}_{j} =\text{z}_{j}\}=D_{j}\text{Z}_{j}+\text{ O}_{j}$$

where $\text{Y}_{j}$ is an $N_{I}N_{K}$*-*element vector of random variables $\left(Y_{ijk}\right)$; $D_{j}$ is an $N_{I}N_{K}\times N_{I}$ matrix of 0s and 1s that translates the $N_{I}$ process errors at time *j* into $N_{I}N_{K}$ process errors at time *j*; $\text{O}_{j}$ is an $N_{I}N_{K}$*-*element vector of random variables generating sampling errors.

Thirdly, we fitted the following state-space model to the simulated set (*s*) of replicated time series of process errors tainted by sampling errors:

eqn 2

$$Z_{ij}=\tau_{j}\sigma_{shared}+\eta_{ij}\sigma_{unshared}$$

where and represent the shared and unshared variations in process errors among sites, respectively. In (eqn 2), $\sigma_{shared}^{2}$ and $\sigma_{unshared}^{2}$represent the amplitude of the shared and unshared fluctuations in process errors among sites, respectively. We computed the intra-class correlation $\hat{\rho}_{ICC}=\frac{\hat{\sigma}_{shared}^{2}}{\hat{\sigma}_{shared}^{2}+\hat{\sigma}_{unshared}^{2}}$ to quantify the strength of the average synchrony among time series of process errors.

Then we repeated steps 1-to-3 200 times with different values of $\bar{\rho}^{proc}$ (0.2, 0.5, 0.8), σ²_s_ (0.25, 1, 4) and $N_{K}$ (5, 10, 20), and compared the estimates $\hat{\rho}_{ICC}$ to $\bar{\rho}^{proc}$ for each parameter combination. Note that in absence of bias, we expect$E_{ps}(\hat{\rho}_{ICC})=\bar{\rho}^{proc}$. We considered the case where σ²_s_ has an assigned value and the case where it is an unknown parameter to be estimated.

Results of the simulations are displayed in Figs. 1-2. Simulations show that the state-space model approach has negligible bias whatever the value of $\bar{\rho}^{proc}$, *σ²_s_* and $N_{K}$, and regardless of whether *σ²_s_* has an assigned value or is an unknown parameter to be estimated.

N_K_=5 N_K_=10 N_K_=20


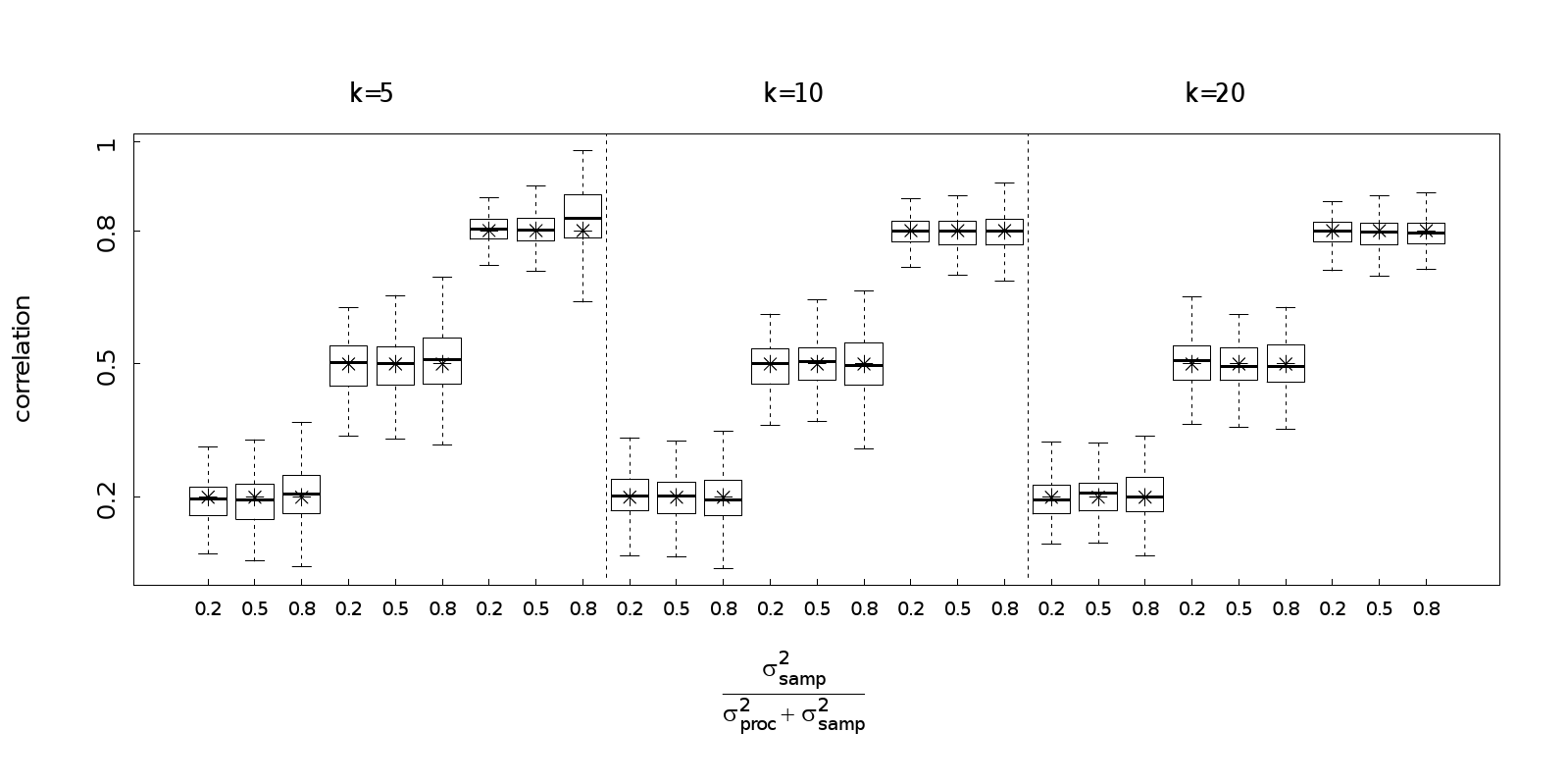


**Figure 1: Results of numerical simulations where σ²_s_ has an assigned value**. The box-plots represent the distribution of the synchrony estimates ($\hat{\rho}_{ICC}$) obtained using our state-space model for different values of the ratio between sampling variance (*σ²_s_*) and the sum of sampling (*σ²_s_*) and process (*σ²_p_*) error variances, and different numbers of replicates ($N_{K}$). Solid black lines represent the average of the 200 estimates of $\rho_{ICC}$ for each parameter combination; the stars represent the corresponding true correlation values ($\bar{\rho}^{proc}$).

N_K_=5 N_K_=10 N_K_=20


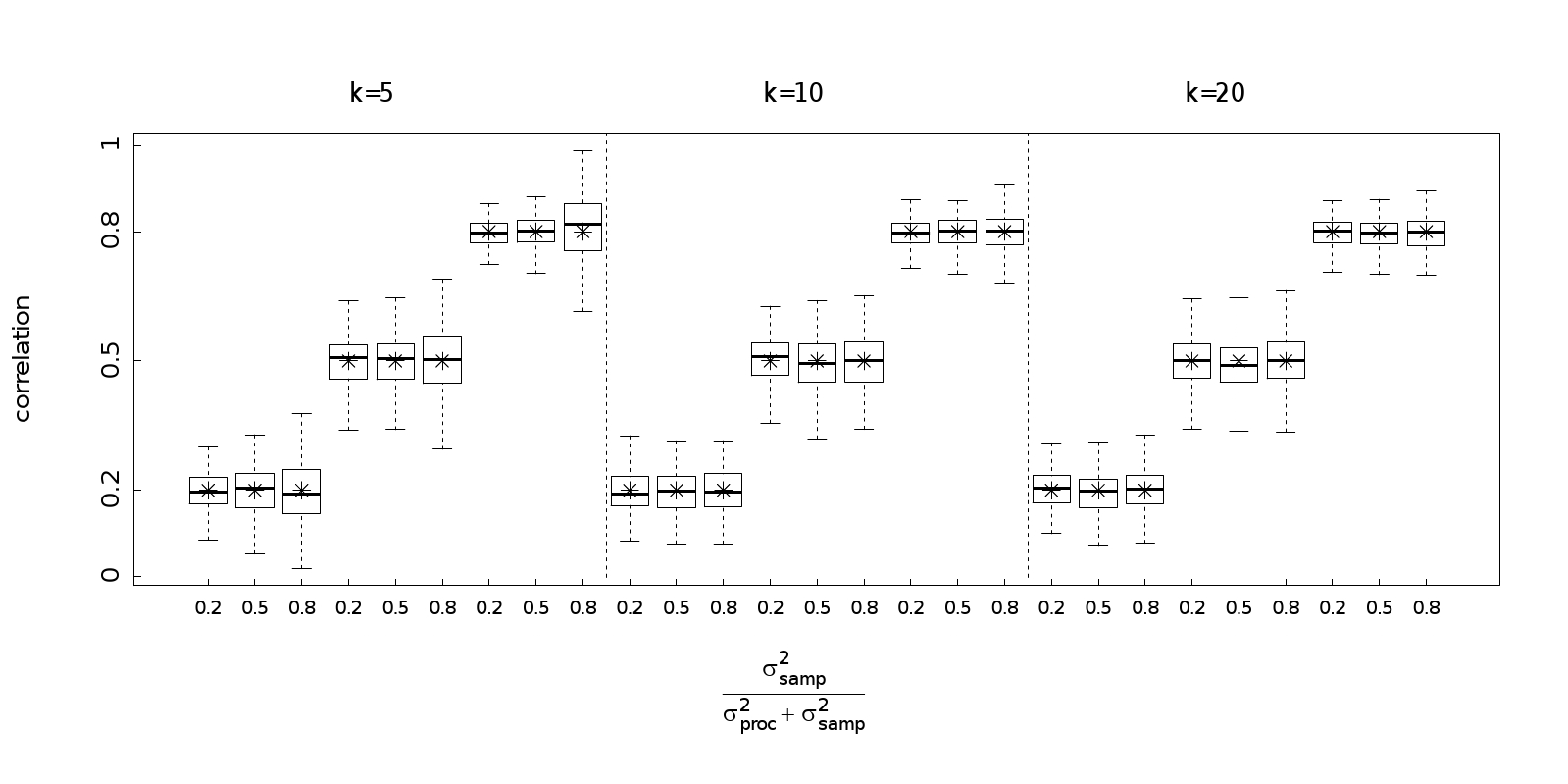


**Figure 2: Results of numerical simulations where *σ²_s_* is considered as an unknown parameter to be estimated.** The box-plots represent the distribution of the synchrony estimates ($\hat{\rho}_{ICC}$) obtained using our state-space model for different value of the ratio between sampling variance (*σ²_s_*) and the sum of sampling (*σ²_s_*) and process (*σ²_p_*) error variances, and different numbers of replicates ($N_{K}$). Solid black lines represent the average of the 200 estimates of $\rho_{ICC}$for each parameter combination; the stars represent the true correlation values ($\bar{\rho}^{proc}$).

**References**

1. Dennis B, Ponciano JM, Taper ML (2010) Replicated sampling increases efficiency in monitoring biological populations. Ecology 91: 610-620.
