# Supplementary material for: Accounting for Sampling Error When Inferring Population Synchrony from Time-Series Data: A Bayesian State-Space Modelling Approach with Applications
Source: PLoS One. 2014 Jan 29;9(1):e87084. doi: 10.1371/journal.pone.0087084 (PMC3906118; doi:10.1371/journal.pone.0087084)
Supplement: File S6 — Detection probability estimates ( ) obtained for the state-space model fitted on the cat dataset. (DOC) [file pone.0087084.s006.doc]

**File S6. Detection probability estimates ($\hat{p}_{ij}$ ) obtained for the state-space model fitted on the cat dataset.**

Posterior mean[95% Credible Interval]

|  | Site | | | |
| --- | --- | --- | --- | --- |
| Time | Port-aux-Français | Port-Jeanne-d'Arc | Port-Couvreux | Ratmanoff |
| 1 | 0.295[0.054;0.845] | - | - | - |
| 2 | 0.317[0.047;0.888] | - | - | - |
| 3 | 0.463[0.075;0.958] | - | - | - |
| 4 | 0.196[0.018;0.676] | 0.598[0.141;0.980] | - | - |
| 5 | 0.634[0.270;0.973] | 0.699[0.244;0.989] | - | 0.195[0.064;0.455] |
| 6 | - | 0.550[0.125;0.972] | - | 0.378[0.101;0.871] |
| 7 | 0.729[0.345;0.988] | 0.595[0.156;0.976] | - | 0.288[0.107;0.638] |
| 8 | 0.761[0.390;0.988] | 0.696[0.247;0.987] | 0.464[0.114;0.941] | 0.606[0.281;0.959] |
| 9 | 0.721[0.348;0.986] | 0.594[0.166;0.977] | 0.446[0.132;0.918] | 0.558[0.200;0.959] |
| 10 | 0.551[0.224;0.947] | 0.668[0.226;0.985] | 0.514[0.140;0.961] | 0.377[0.152;0.790] |
| 11 | 0.565[0.213;0.965] | 0.409[0.051;0.947] | 0.377[0.079;0.901] | 0.575[0.235;0.962] |
| 12 | 0.542[0.213;0.948] | 0.681[0.237;0.986] | 0.451[0.146;0.919] | 0.427[0.181;0.836] |
| 13 | 0.756[0.351;0.991] | 0.582[0.148;0.977] | 0.490[0.119;0.952] | 0.615[0.276;0.966] |
| 14 | 0.500[0.160;0.936] | 0.675[0.224;0.986] | 0.733[0.310;0.989] | 0.492[0.216;0.885] |
| 15 | 0.688[0.293;0.984] | 0.629[0.187;0.981] | - | 0.672[0.344;0.972] |
| 16 | 0.598[0.223;0.971] | 0.490[0.091;0.963] | - | 0.354[0.145;0.735] |
| 17 | 0.323[0.031;0.892] | 0.427[0.054;0.953] | - | 0.468[0.200;0.878] |
| 18 | - | 0.678[0.225;0.986] | 0.704[0.256;0.987] | 0.719[0.334;0.987] |
| 19 | - | 0.641[0.185;0.983] | - | 0.737[0.365;0.987] |
| 20 | 0.660[0.257;0.980] | 0.653[0.198;0.983] | - | 0.701[0.338;0.981] |
| 21 | 0.317[0.105;0.673] | 0.648[0.197;0.983] | - | 0.714[0.345;0.984] |
| 22 | 0.737[0.327;0.989] | 0.662[0.204;0.984] | 0.620[0.180;0.979] | 0.473[0.215;0.807] |
| 23 | - | 0.638[0.179;0.983] | 0.646[0.207;0.981] | 0.672[0.282;0.980] |
